# An Improved PDE6D Inhibitor Combines with Sildenafil To Inhibit *KRAS* Mutant Cancer Cell Growth

**DOI:** 10.1021/acs.jmedchem.3c02129

**Published:** 2024-05-17

**Authors:** Pelin Kaya, Elisabeth Schaffner-Reckinger, Ganesh babu Manoharan, Vladimir Vukic, Alexandros Kiriazis, Mirko Ledda, Maria Burgos Renedo, Karolina Pavic, Anthoula Gaigneaux, Enrico Glaab, Daniel Kwaku Abankwa

**Affiliations:** † Cancer Cell Biology and Drug Discovery Group, Department of Life Sciences and Medicine, University of Luxembourg, 4365 Esch-sur-Alzette, Luxembourg; ‡ Faculty of Technology, University of Novi Sad, 21000 Novi Sad, Serbia; § Turku Bioscience Centre, University of Turku and Åbo Akademi University, 20520 Turku, Finland; ∥ Luxembourg Center for Systems Biomedicine, University of Luxembourg, 4365 Esch-sur-Alzette, Luxembourg; ⊥ Bioinformatics Core, Department of Life Sciences and Medicine, University of Luxembourg, 4365 Esch-sur-Alzette, Luxembourg

## Abstract

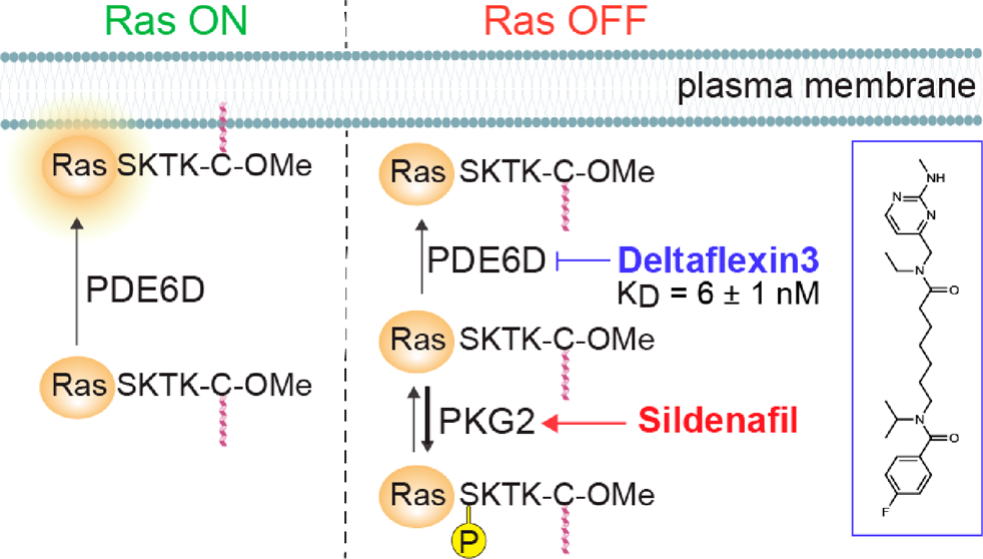

The trafficking chaperone PDE6D (or PDEδ) was proposed as a surrogate target for K-Ras, leading to the development of a series of inhibitors that block its prenyl binding pocket. These inhibitors suffered from low solubility and suspected off-target effects, preventing their clinical development. Here, we developed a highly soluble, low nanomolar PDE6D inhibitor (PDE6Di), Deltaflexin3, which has the lowest off-target activity as compared to three prominent reference compounds. Deltaflexin3 reduces Ras signaling and selectively decreases the growth of *KRAS* mutant and *PDE6D*-dependent cancer cells. We further show that PKG2-mediated phosphorylation of Ser181 lowers K-Ras binding to PDE6D. Thus, Deltaflexin3 combines with the approved PKG2 activator Sildenafil to more potently inhibit PDE6D/K-Ras binding, cancer cell proliferation, and microtumor growth. As observed previously, inhibition of Ras trafficking, signaling, and cancer cell proliferation remained overall modest. Our results suggest reevaluating PDE6D as a K-Ras surrogate target in cancer.

## Introduction

The highly mutated oncogene *KRAS* is one of the best-established cancer targets. Only recently have two *KRAS-G12C* inhibitors, sotorasib and adagrasib, been approved for the treatment of lung cancer.^1,2^ While other allele-specific, pan-Ras, and Ras pathway inhibitors are under intense development,^3,4^ there is still a need to target Ras more profoundly from various angles.

Inhibition of Ras membrane targeting remains a promising strategy for inhibitor development.^5,6^ The trafficking chaperone PDE6D (or PDEδ) has been proposed as a surrogate drug target in *KRAS* mutant cancers.^7^ PDE6D possesses a hydrophobic pocket, which can bind to one or even two prenyl moieties, thus having a cargo spectrum that comprises farnesylated or geranylgeranylated Ras and Rho family proteins as well as Rab proteins.^8,9^ Only proteins that are not in addition palmitoylated in the vicinity of the prenylated cysteine are accepted as cargo, making mono- and dual-palmitoylated N-Ras, K-Ras4A, and H-Ras effectively worse PDE6D cargo in cells than K-Ras4B (hereafter K-Ras).^10^ Cargo affinity is critically modulated by the four residues upstream of the prenylated cysteine. Structure and sequence comparisons suggest that the two residues immediately upstream of the prenylated cysteine cannot be large amino acids, like Lys, Arg, or Glu.^8^ This stretch of four residues also comprises Ser181 at the C-terminus of K-Ras, which can be phosphorylated by PKG2.^11^ Binding data of PDE6D to K-Ras with a S181E mutation suggest a reduced interaction when K-Ras is phosphorylated on Ser181.^8^

K-Ras has only micromolar affinity to PDE6D, while another cargo, the inositol phosphatase INPP5E, has a low nanomolar affinity.^8,12^ This has important consequences for their subcellular distribution. While K-Ras can be released in the perinuclear area by the allosteric release factor Arl2, which binds to PDE6D when GTP bound,^13,14^ INPP5E is only dislodged by GTP-Arl3 inside the primary cilium.^12^

The development of inhibitors that competitively bind to the prenyl pocket of PDE6D was pioneered by the Waldmann group.^15^ However, their first two generations of PDE6D inhibitors (PDE6Di) Deltarasin and Deltazinone1 appeared to have off-target issues and poor metabolic stability, respectively.^7,16^ In addition, both compounds were ejected by the GTP-Arl2-dependent mechanism, similar to natural PDE6D cargo. Only their third-generation inhibitors, the Deltasonamides, could withstand GTP-Arl2-mediated ejection as they were highly optimized for subnanomolar affinity. However, these compounds appeared to have low cell penetration.^15^ In an attempt to optimize the pharmacological properties, the chemotype was switched from benzimidazole to pyridazinones, such as Deltazinone.^16^ This led to the development of low nanomolar inhibitors, such as candidate compound **99** that was pharmacokinetically evaluated in mice, without assessment of antitumorigenic activity.^17^ Hence, from these pioneering compounds, antitumor activity in vivo was only demonstrated with the first-generation compound Deltarasin.^7^ All three compound generations were mostly evaluated in *KRAS* mutant pancreatic cancer cell lines, yet both Deltarasin and Deltasonamide were also active in micromolar amounts in *KRAS* mutant and *PDE6D*-dependent colorectal cancer cell lines.^18^

Another class of more recent PDE6Di is proteolysis targeting chimeras (PROTACs). Unlike classical competitive inhibitors, they do not have to bind permanently, i.e., they can act substoichiometrically.^19^ Proof-of-concept PROTACs from two groups were developed based on previously established competitive PDE6Di, Deltasonamide and Deltazinone.^20,21^ These heterobifunctional compounds bind with their first functional moiety to the prenyl pocket of PDE6D, and with the second they recruit an E3 ubiquitin ligase complex to instruct proteasomal degradation of PDE6D. While the Deltasonamide-derived PROTAC effectively decreased PDE6D levels in pancreatic cancer cells,^20^ the Deltazinone-derived PROTAC was even efficacious in SW480 xenografts in mice.^21^

Following the pioneering work of the Waldmann group, other PDE6D pocket competitive inhibitors were investigated, although for several of them clear in vitro or cellular target engagement data are missing. However, the Sheng group developed compounds that bound to PDE6D in vitro with nanomolar affinity. Some suppressed MAPK output but again had only micromolar cellular activity.^22,23^ Interestingly, in their most recent work, their spirocyclic compound **36l** (*K*_D_ = 127 nM) showed target engagement in cells while also demonstrating in vivo efficacy against *KRAS* mutant primary cell line-derived xenografts.^24^ In another study, compounds around a triazole scaffold were developed, of which compound **27** had nanomolar activity in a PDE6D binding assay and robustly inhibited MAPK output at 10 μM and A549 cell growth at this concentration range.^25^

Another PDE6Di emerged from a Rac-inhibitor screen, which led to the oxadiazole DW0254 as a submicromolar active compound (*K*_D_ = 436 ± 6 nM).^26^ This compound inhibited downstream signaling of Ras above 20 μM, and in vivo activity was observed with pretreatment of the transplanted T-cell cancer cell line or subcutaneous implantation of an osmotic pump due to limited solubility.

We have previously published novel competitive PDE6Di called Deltaflexins, for which we determined low micromolar affinities in a dedicated surface plasmon resonance assay, which were matched by a similar level of activity in *KRAS* mutant HCT116 and MDA-MB-231 cancer cells.^27^ Their chemical design features a hexamethylene–amide backbone, which allowed simple derivatization and compound evolution. Importantly, Deltaflexins demonstrated the expected K-Ras over H-Ras selectivity in cells, an important on-target feature.

A number of questions remain unresolved regarding PDE6D as a surrogate target for K-Ras. Current PDE6Di are still at the hit stage and have various problems, such as poor solubility, metabolic instability, and off-target issues.^16,17^ This makes the interpretation of phenotypic data and validation of PDE6D as a drug target in vivo difficult.^7^ Together with the broad cargo spectrum of PDE6D, which involves far more prenylated proteins than K-Ras, it is almost impossible to tell in which cancer type PDE6Di should be applied. Hence, clear genetic determinants that could indicate a susceptibility to PDE6D inhibition are lacking.

Here, we established an in silico library of compounds by cross-hybridizing moieties of existing PDE6Di with our previous hexamethylene–amide backbone.^27^ Aided by computational docking, we derived rationales for the synthesis of 16 novel PDE6Di that we comprehensively characterized biochemically and in cells for potency and K-Ras and PDE6D on-target selectivity. We demonstrate that increased efficacy and more focused inhibition of K-Ras can be achieved by combining our most selective and highly soluble PDE6Di Deltaflexin3 with the clinically approved Sildenafil.

## Results

### Computational Docking Aided Design of Novel PDE6D Inhibitors

We previously demonstrated that PDE6Di can be efficiently generated by using a hexamethylene–amide backbone.^27^ Using this backbone as a base, we created an in silico library of hybrid compounds, which also contained moieties of established PDE6Di and Salirasib (farnesylthiosalicylic acid), which was included due to its similarity with PDE6D binders (Data S1).^9,28^

**Figure 1:**
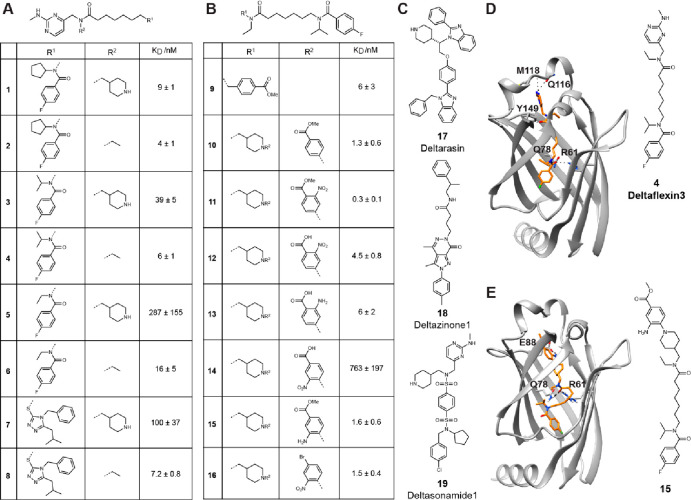
Investigated PDE6Di with affinities and computational docking. (A and B) Structures of developed first (A) and second (B) round PDE6Di with PDE6D dissociation constants (*K*_D_) measured using F-Ator in a fluorescence polarization assay; *n* ≥ 2. (C) Chemical structures of employed reference PDE6Di molecules. (D and E) Computational docking pose of Deltaflexin3 (**4**) (D) and **15** (E) to PDE6D in its open state (PDB ID 4JV8). Putative hydrogen bonds with indicated residues are shown as dashed lines.

Altogether, 305 compounds were thus designed in the first round and computationally docked to human PDE6D (PDB ID 4JV8) using Glide docking software.^29^ This structure was chosen because among the publicly available crystal structures it has one of the highest resolutions (1.45 Å) and is in complex with a bound inhibitor, i.e., in a conformationally open state where the prenyl binding pocket is accessible. Compounds selected based on the docking scores, MM-GBSA binding energy, and visual inspection were prioritized and provided a rationale for the synthesis of a first round of eight compounds that were biochemically and cell-biologically characterized (Figure 1A; Data S2 and S3).

Subsequently, the best-performing compound **4**, hereafter Deltaflexin3, was chosen as a starting point for derivatives that were again first evaluated by in silico docking using SeeSAR software. In this second round, compounds were extended to attempt interactions with residues at the entry of the hydrophobic pocket of PDE6D. Based on these computational data, a second round of eight candidate compounds was synthesized and characterized like the first-round compounds (Figure 1B; Data S2 and S3). In order to properly benchmark our compounds, we employed previous PDE6Di Deltarasin, Deltazinone1, and Deltasonamide1 as references in this study (Figure 1C).^7,15,16^

Computational docking data of two of our compounds, Deltaflexin3 and **15**, revealed multiple van der Waals contacts to residues Met20, Arg61, Gln78, and Tyr149. Hydrogen bonds were predicted for Deltaflexin3 with Arg61, Gln78 at the base of the pocket, and Gln116, Met118, and Tyr149 at the pocket entry (Figure 1D). By contrast, predicted hydrogen bonds of **15** were limited to Arg61, Gln78 at the base, and Glu88 at the top of the pocket (Figure 1E). The Arg61 and Tyr149 hydrogen bonds are shared with those identified for Deltarasin and predicted for Deltazinone1.^7,16^

### In Vitro Affinity and Intracellular BRET Assays Quantify Target Engagement and K-Ras Selectivity

**Figure 2:**
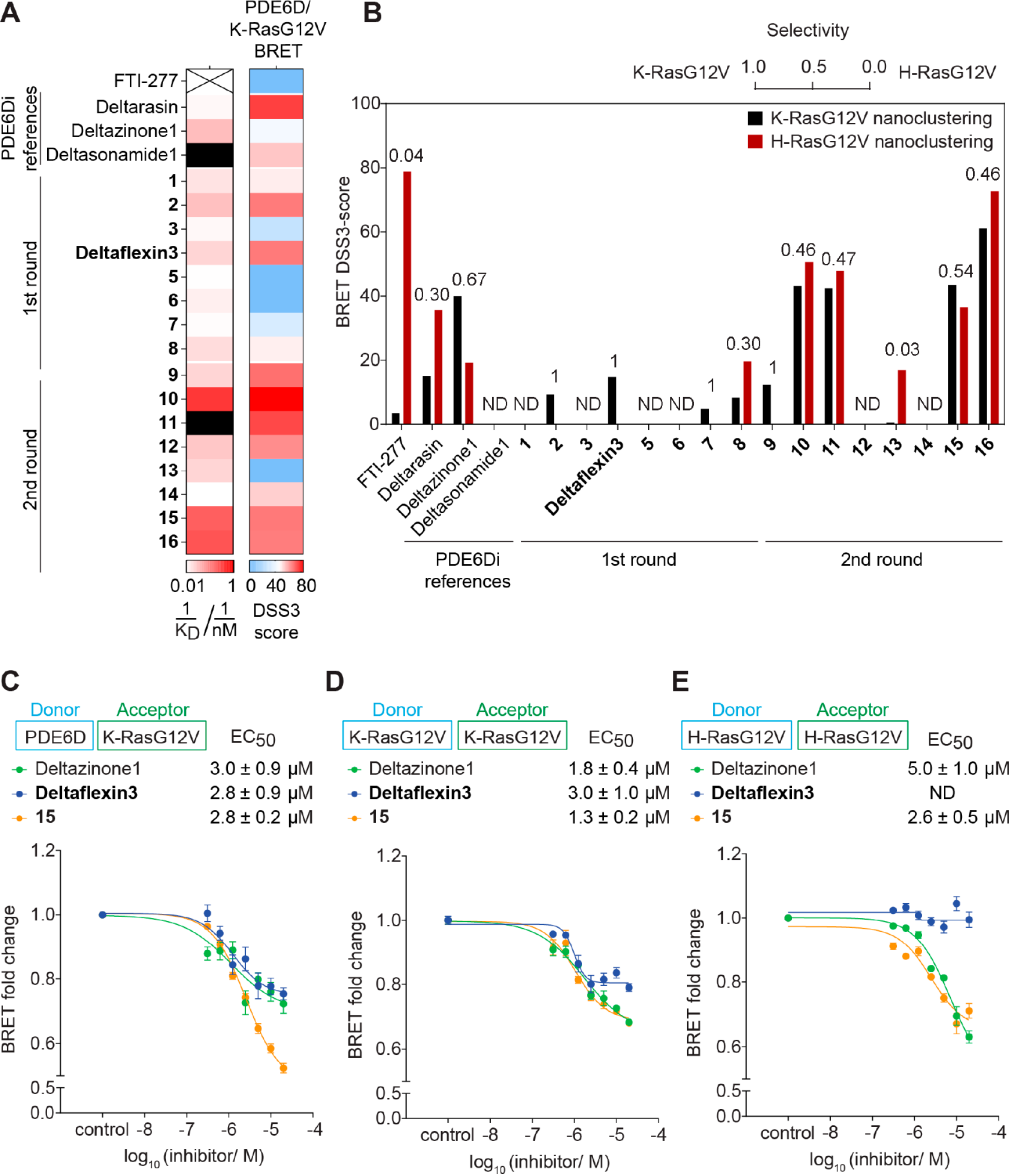
Quantification of on-target activity of PDE6Di in vitro and in cellular BRET assays. (A) Heatmaps of in vitro affinity of compounds determined using F-Ator (first column; *n* ≥ 2), and DSS3 scores from cellular BRET experiments (second column; *n* ≥ 2). The disruption of the PDE6D/K-RasG12V complex was measured by BRET over a wider concentration range, and the area under the curve DSS3 score was determined. (B) Quantification of K-RasG12V selectivity (values above bars) was performed by determining the ratio of K-RasG12V and the sum of K-RasG12V- and H-RasG12V-BRET DSS3 scores; *n* ≥ 2. (C–E) Dose-dependent change of normalized BRET signals after treatment with indicated compounds using the BRET donor/acceptor pairs shown on top; *n* ≥ 4.

All 16 compounds that were prioritized for synthesis first underwent in vitro testing using a previously employed fluorescence polarization assay where the FITC-labeled PDE6D binder Atorvastatin (F-Ator) was used as a probe, as common in other PDE6Di studies^7^ (Figure 1A and 1B; Data S3). In addition, we determined the affinities of compounds using the FITC-labeled farnesylated peptide derived from the C-terminus of the small GTPase Rheb (F-Rheb)^14^ (Data S3). When using F-Ator as a probe, we recovered affinities in the low nanomolar range for reference compounds, Deltarasin (*K*_D_ = 39 ± 15 nM), Deltazinone1 (*K*_D_ = 3.8 ± 0.4 nM), and Deltasonamide1 (*K*_D_ = 0.11 ± 0.03 nM), similar to previously published values.^7,15,16^ By contrast, affinities determined using F-Rheb were typically only in the submicromolar range (Data S3). However, both data sets overall correlated and served to rank the in vitro potencies of our 16 compounds, and in the following, we will refer to the values obtained with F-Ator unless otherwise stated (Figure 2A, Figure S1A).

Subsequently, three cellular BRET (bioluminescence resonance energy transfer) assays were applied to profile the disruption of the PDE6D/K-Ras interaction and loss of functional membrane organization of K-Ras as compared to H-Ras over a wider concentration range in HEK293-EBNA cells. In analogy to our previous FRET-based target engagement assay,^27^ we implemented a BRET assay with Rluc8-PDE6D and GFP2-K-RasG12V to determine the intracellular potency of compounds to displace K-RasG12V from PDE6D (Figure 2A, Figure S1B).

While intracellular EC_50_ values were in the micromolar regime (Data S3), we generally employed the more robust normalized area under the curve DSS3 score for dose–response data.^30^ Overall, DSS3 scores from the PDE6D/K-RasG12V-BRET correlated with in vitro affinities, and in both data sets, potencies increased markedly from the first to the second round of compounds (Figure 2A).

A second set of BRET assays was likewise built in analogy to previous FRET assays.^31,32^ We assessed the BRET that emerges between a Rluc8-donor-tagged RasG12V and a GFP2-acceptor-tagged RasG12V due to nanoclustering.^33^ This type of assay can sensitively detect perturbations not only of Ras nanoclustering but also of any upstream process, such as correct membrane anchorage or lipid modifications^33,34^ (Figure S1C–F).

The large dynamic range of this BRET assay is illustrated by data reporting the effect of Mevastatin treatment or the combination of a farnesyl-transferase inhibitor with a geranylgeranyl-transferase I inhibitor, which led to a 70–80% reduction of the signal. Moreover, the signal-to-noise ratio, which is the ratio of the values of the control and the Mevastatin treatment, was 4.8 in the BRET assay (Figure S1F). It compared favorably to the microscopy-based analysis of the plasma membrane to cytoplasm signal ratios, which albeit suggesting similar effect trends has a signal-to-noise ratio of only 1.6 (= 5.2 [ratioPM/cytoplasm _control_]/3.3 [ratioPM/cytoplasm _Mevastatin_]) and inherently more noise in the source data (Figure S2).

When palmitoylated, prenylated proteins such as dually palmitoylated H-Ras cannot bind to PDE6D, making them effectively worse intracellular cargo.^8,10^ Hence, loss of PDE6D activity such as by siRNA-mediated knockdown, selectively decreases the BRET signal of K-RasG12V, but not of H-RasG12V (Figure S1C–E).

Using these two BRET assays, we assessed the intracellular K-RasG12V membrane anchorage disruption and K-RasG12V selectivity of compounds. This again revealed an increase in potency among the second-round compounds (Figure 2B). Deltaflexin3 had the best overall K-RasG12V selectivity, compound **15** had the best selectivity of the top second-round compounds (Figure 2B), and both compounds compared well in all three BRET assays relative to the most selective reference compound Deltazinone1 (Figure 2C–E).

### Assessing the off-Target Activity of the Top Compounds

Despite clearly inhibiting PDE6D, several compounds including reference compounds Deltarasin and Deltazinone1 did not display exclusive K-RasG12V selectivity (Figure 2B). This may be due to off-target activities, a problem that was already noted for previous PDE6Di by others.^16,17^

**Figure 3:**
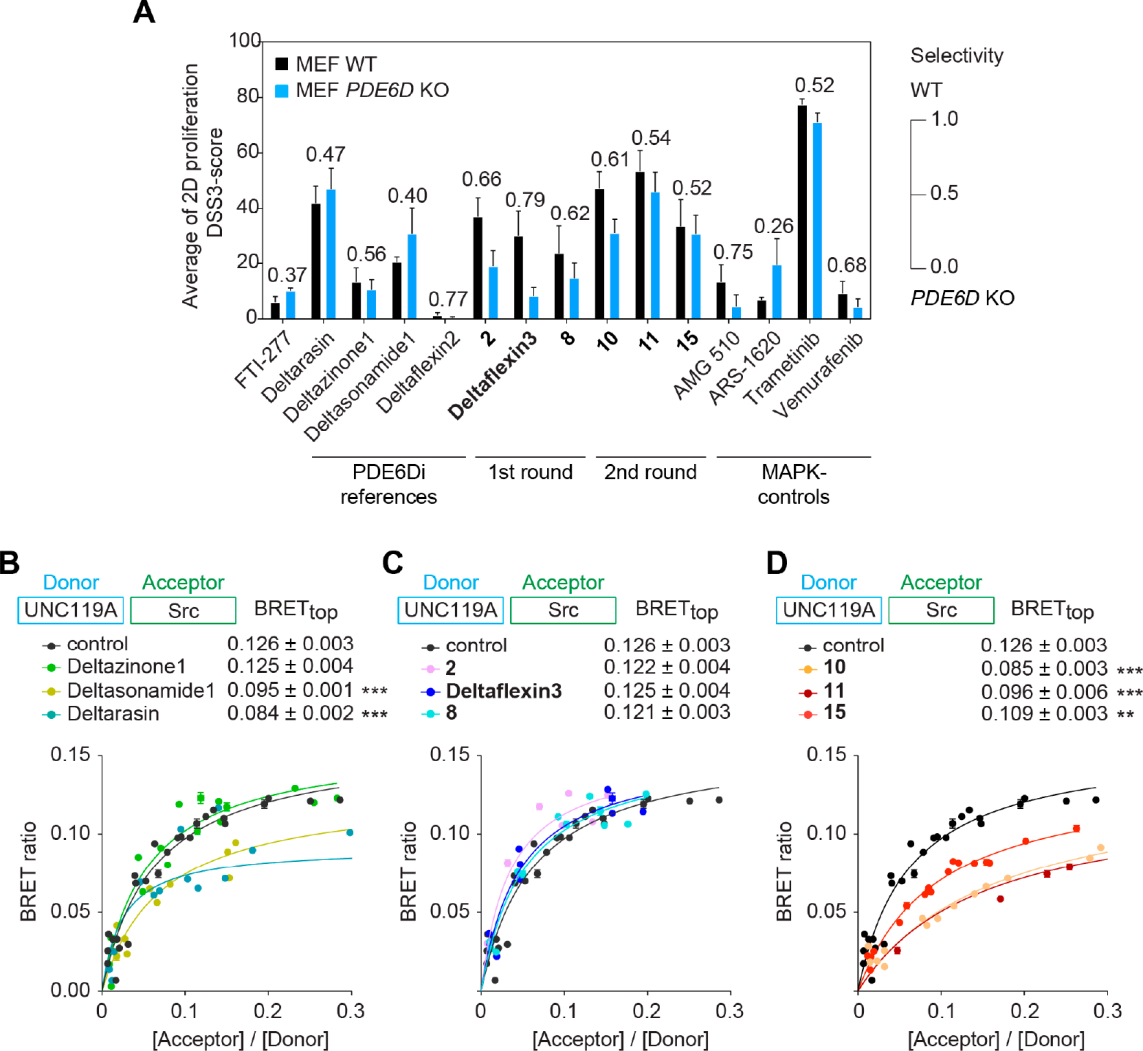
Analysis of PDE6Di off-target activities. (A) DSS3 scores of indicated compounds from 2D proliferation assays acquired with WT or *PDE6D* KO MEFs; *n* = 4. PDE6D selectivity was determined as the ratio of the DSS3 scores from WT and the sum of WT and KO MEFs and is indicated above the bars. (B–D) BRET titration curves of UNC119A/Src complex after treatment with indicated reference PDE6Di (B) and top first-round (C) or top second-round (D) compounds at 5 μM; *n* ≥ 3. Statistical comparisons of BRET_top_ values to controls were done using two-tailed Student’s *t* test.

Broad off-target effects are phenotypically determined by comparing the antiproliferative effect of compounds on cells with and without the target. We therefore compared the cell growth inhibition of MEF cells with a homozygous CRISPR-mediated knockout (KO) of *PDE6D* to their wild-type (WT) counterpart as a measure of PDE6D selectivity^35^ (Figure S3A). In line with the BRET-derived K-RasG12V selectivity data (Figure 2B; Figure S3B), first-round compounds exhibited a higher PDE6D selectivity than second-round compounds, with Deltaflexin3 showing again the highest overall selectivity (Figure 3A).

UNC119A is a trafficking chaperone of myristoylated proteins and structurally homologous to PDE6D.^12^ Given this relatedness in structure and function, it is a plausible off-target for PDE6Di. We therefore established a BRET assay to determine the UNC119A-directed off-target activity by quantifying if the top three compounds from each round disrupted the UNC119A/Src complex.

In BRET titration experiments, the characteristic BRET ratio, BRET_top_, which is reached within a defined acceptor-to-donor ratio, is a measure for complex stability.^36^ A previously identified inhibitor of UNC119A, Squarunkin A, significantly reduced BRET_top_ between UNC119A-Rluc8 and Src-GFP2 (Figure S3C).^37^ Similarly, treatment with the *N*-myristoyl-transferase inhibitor IMP-1088 reduced BRET_top_ (Figure S3C),^38^ confirming that our assay can detect myristoyl pocket-dependent disruption of the UNC119A/Src interaction.

When testing the reference compounds, we found that surprisingly at 5 μM both Deltarasin and Deltasonamide1, but not Deltazinone1, significantly decreased the UNC119A/Src-BRET, suggesting off-target binding of these two compounds to UNC119A (Figure 3B). By contrast, none of our top first-round compounds decreased UNC119A/Src-BRET (Figure 3C), while all our top second-round compounds did, with **15** having the least disruptive activity (Figure 3D).

### Inhibition of Ras Signaling and Cancer Cell Proliferation by the Top Compounds

**Figure 4:**
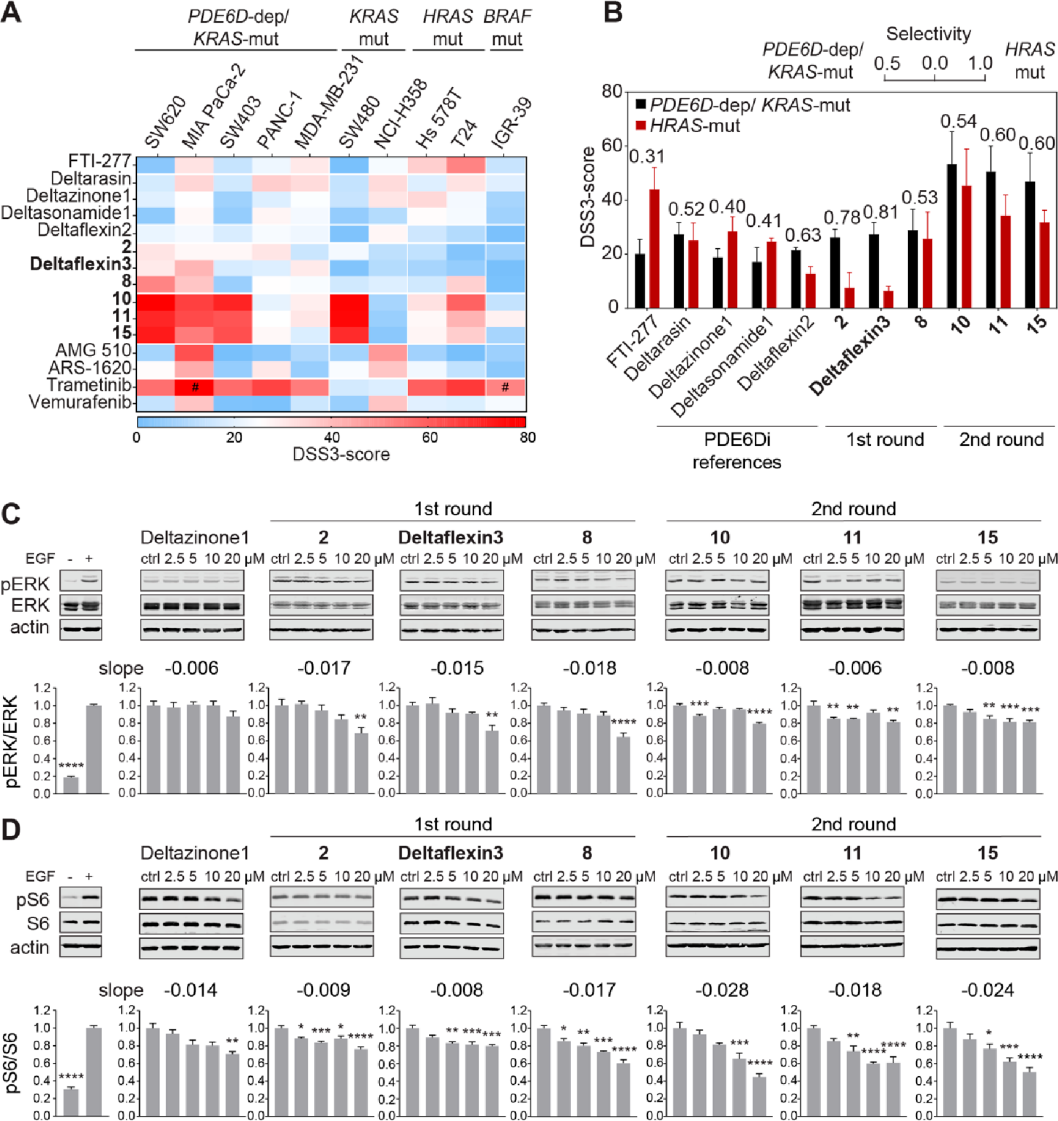
Inhibition of cell proliferation and Ras signaling by PDE6Di. (A) DSS3 scores of indicated compounds from 2D proliferation assays acquired with *PDE6D*-dependent and *KRAS* mutant, *KRAS* mutant, *HRAS* mutant, or *BRAF* mutant cell lines; *n* ≥ 2; (#) *n* = 1. (B) Quantification of *PDE6D*-dependent and *KRAS* mutant selectivity was performed by determining the ratio of the average of DSS3 scores from *PDE6D*-dependent and *KRAS* mutant cell lines and the sum of the former and the average DSS3 score of *HRAS* mutant cell lines from A; *n* ≥ 3, except for the condition T24/compound **8**, where *n* = 2. (C and D) Cropped example images with quantification of immunoblot data of phosphorylated and total ERK (C; *n* ≥ 4) or phosphorylated and total S6 (D; *n* ≥ 4, except *n* [compound **8**] = 3) from *KRAS-G12C* mutant MIA PaCa-2 cells treated with indicated compounds for 4 h before EGF stimulation; stimulation control data to the far left. Slope values of the linear fits to the treatment–response data are given. Statistical differences to control samples were determined by employing one-way ANOVA with Dunnett’s multiple comparisons test.

Next, we continued our selectivity assessment by testing the antiproliferative activity of the top three compounds from each round on *KRAS*, *HRAS*, or *BRAF* mutant cancer cells. In line with in vitro and BRET data (Figure 2A and 2B), the antiproliferative activity was significantly increased in compounds of the second optimization round with cellular potencies increasing to the low- and submicromolar regime (Figure 4A; Data S3) but at the expense of selectivity (Figure 4B).

By contrast, Deltaflexin3 displayed the overall highest selectivity for *PDE6D*-dependent and *KRAS* mutant, as compared to *HRAS* mutant cancer cell lines (Figure 4B; Figure S3D), consistent with its K-RasG12V selectivity detected by BRET (Figure 2B) and its off-target activity being lowest among the investigated compounds (Figure 3A and 3C). It therefore surpassed the most selective reference compound, Deltazinone1. The highest activity of Deltaflexin3 was seen in MIA PaCa-2 (*KRAS-G12C* mutant) cells (EC_50_ = 6 ± 1 μM; Data S3), in line with the high *KRAS* and *PDE6D* dependence of this cell line (Figure S3D).^39^

For compounds that significantly disrupt K-RasG12V membrane anchorage, it is expected that they also reduce Ras-signaling output. In line with previous data,^7,16^ the reduction in phospho-ERK (Figure 4C) and phospho-S6 levels (Figure 4D) downstream of Ras was modest in MIA PaCa-2 cells upon treatment with our top compounds but better than that seen with the overall best reference compound Deltazinone1.

We subsequently focused our analysis on Deltaflexin3 given its overall best performance across all assays and its high PBS solubility (kinetic solubility, *S* = 1.9 mg/mL), which compares favorably to Deltazinone1 (kinetic solubility, *S* = 0.008 mg/mL), the most soluble compound among the reference compounds.

### PDE6D Inhibitor Deltaflexin3 and Sildenafil Combine To Inhibit K-Ras Activity

The approved drug Sildenafil is an inhibitor of cGMP-specific phosphodiesterase type 5 (PDE5), thus increasing cGMP levels. It stimulates the cGMP-dependent PKG2, which phosphorylates Ser181 on the C-terminus of K-Ras.^11^ Given that the phospho-mimetic K-Ras-S181E mutation was shown to reduce the affinity to PDE6D ∼6-fold,^8^ we reasoned that Sildenafil treatment would likewise decrease the affinity.

We therefore sought to increase the antitumorigenic activity of Deltaflexin3 by combining it with Sildenafil, which would also focus the inhibitory activity on K-Ras. A more focused inhibition is supported by a survey of >150 small GTPases, which suggests that only 15 other established or predicted PDE6D cargo proteins possess serine or threonine residues in the four-residue stretch upstream of the prenylated cysteine that could be affected by Sildenafil in a manner that could impact PDE6D engagement (Data S4).

**Figure 5:**
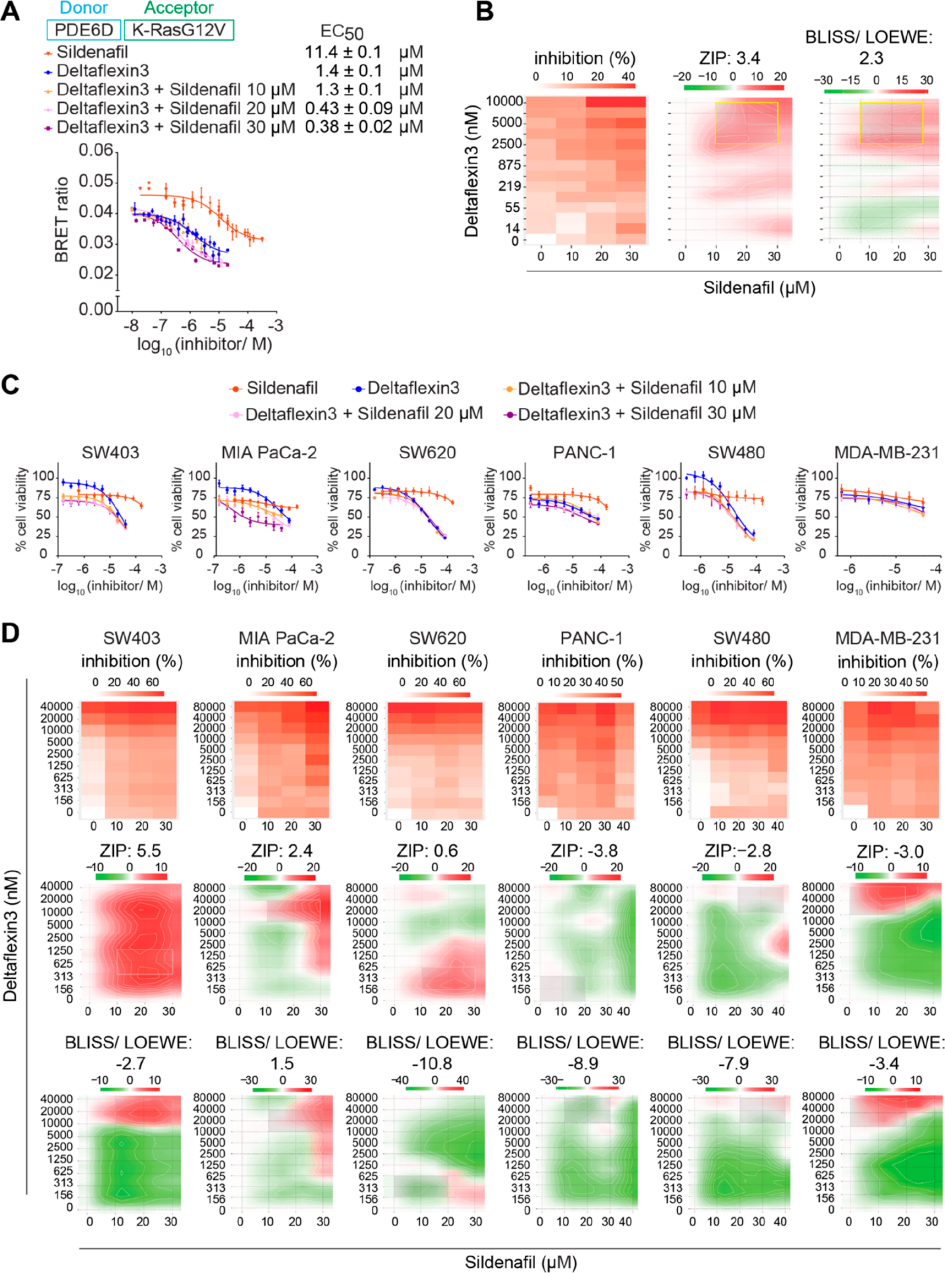
Analysis of Deltaflexin3 and Sildenafil synergism. (A) Dose-dependent disruption of PDE6D/K-Ras complex after treatment with indicated compounds and combinations measured in cellular BRET assays; *n* ≥ 3. (B) Inhibition (drop in normalized BRET ratio, left), ZIP (middle), and BLISS/LOEWE (right) synergism heatmaps of combinations shown in A. Positive synergy scores indicate synergism, while negative scores signify antagonism. The concentration regimes with high synergism of interest are boxed in yellow. (C) Compound–dose-dependent change of cell proliferation after indicated treatments of *KRAS* mutant cancer cell lines; *n* ≥ 2. (D) Inhibition (top), ZIP (middle), and BLISS/LOEWE (bottom) synergism heatmaps for combinatorial Deltaflexin3 and Sildenafil treatment as determined from 2D cell proliferation assays shown in C. Faint gray boxes mark areas of interest suggested by the algorithm. BLISS/LOEWE synergy scores < −5 indicate false positive synergy.

Using our PDE6D/K-RasG12V-BRET assay, we found that indeed Sildenafil dose dependently reduced the BRET signal consistent with a disruption of the PDE6D/K-RasG12V complex (EC_50_ = 11.4 μM) (Figure 5A).

Given that the BRET biosensor had to be expressed with the K-RasG12V acceptor construct in excess for an optimal dynamic range, the response range of the Sildenafil curve appeared upward shifted as compared to the Deltaflexin3 curve (Figure 5A). The same was observed with manipulations that block K-RasG12V prenylation (Figure S3E).

We then combined Deltaflexin3 with Sildenafil at 10, 20, and 30 μM, i.e., concentrations in the pseudolinear regime of the Sildenafil dose–response curve (Figure 5A). This analysis revealed high synergy scores with both the default ZIP model and the most stringent BLISS/LOEWE consensus model (Figure 5B). Areas with high synergism can be found at concentrations of ∼20 μM Sildenafil and ∼2.5–10 μM Deltaflexin3 (Figure 5B).

We therefore continued with a 2D proliferation analysis for synergism in five *KRAS* mutant and dependent cancer cell lines with diverse levels of *PDE6D* and *PRKG2* dependencies (Figure 5C, Figure S3D). Among the tested cell lines, MIA PaCa-2 and SW403 show the highest ZIP synergy scores, but only MIA PaCa-2 shows a clear shift of the inhibition curve to lower concentrations for combinations of the drugs and also has a high BLISS/LOEWE consensus synergy score, which is the most stringent score developed to exclude false positives (Figure 5D).^40^ Both cell lines show a sensitivity to *PDE6D* and antisensitivity to *PRKG2* (Figure S3D), which is consistent with their increased response to the drug combination. Importantly, high synergism was observed at similar concentrations that were identified using the on-target BRET assay (Figure 5B and 5D).

### Combinations of Deltaflexin3 and Sildenafil Efficiently Suppress Ras Signaling and Microtumor Growth

Supported by these proliferation data that suggested a synergism of Deltaflexin3 in combination with Sildenafil, we focused our investigations on MIA PaCa-2 cells.

**Figure 6:**
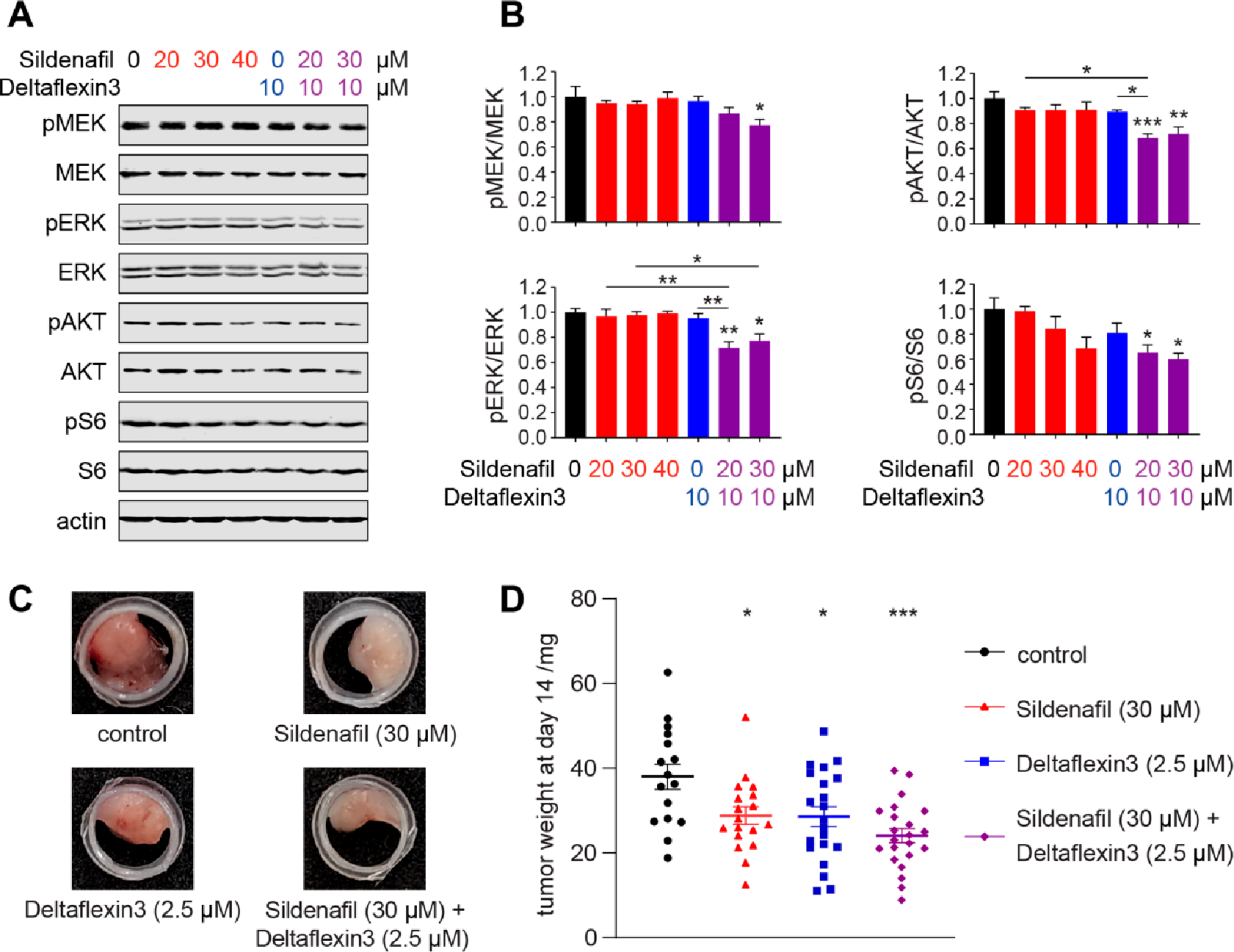
The Deltaflexin3/Sildenafil combination more potently inhibits Ras signaling and microtumor growth. (A) Cropped example images of immunoblot data of lysates from *KRAS-G12C* mutant MIA PaCa-2 cells treated with indicated compounds for 4 h before EGF-stimulation. Analyzed signaling proteins are explained in the main text. Actin served as loading control. (B) Quantification of repeats from immunoblot data of phosphorylated and total MEK, ERK, AKT, and S6 (*n* = 4). Asterisks directly above the error bars indicate statistical significance as compared to the nontreated control condition. (C) Representative images of microtumors formed by MIA PaCa-2 cells grown in the CAM assay and treated with inhibitors as indicated. (D) Weights of the MIA PaCa-2-derived microtumors (≥16 per condition from *n* = 5) after treatment with Deltaflexin3 or/and Sildenafil.

We first reexamined whether signaling downstream of Ras was more efficiently inhibited by the combination treatment. Neither Sildenafil at concentrations between 20 and 40 μM nor Deltaflexin3 at 10 μM significantly reduced phosphorylated (p) ERK, pMEK, pAkt, or pS6 levels (Figure 6A and 6B). In line with the previously observed synergism in the BRET assay (Figure 5A and 5B), the combination of 10 μM Deltaflexin3 and 20 or 30 μM Sildenafil significantly reduced normalized phosphorylation levels of all aforementioned signaling proteins (Figure 6A and 6B).

Next, we evaluated the antitumorigenic activity of Deltaflexin3 also in combination with Sildenafil. While Deltaflexin3 at 10 mg/kg/d alone slowed tumor growth in an MDA-MB-231 mouse xenograft model (Figure S4A), these results were not significant as compared to the analogous setup in the chorioallantoic membrane (CAM) assay (Figure S4B). In the CAM assay, microtumors are raised on the chorioallantoic membrane of fertilized chick eggs.^41,42^ This assay allows a simpler compound application and has lesser demands on the formulation. We therefore continued our evaluation for the antitumorigenic activity in the CAM assay.

While 10 μM Deltaflexin3 alone significantly reduced MDA-MB-231 cell-derived microtumors (Figure S4B), already 2.5 μM Deltaflexin3 was sufficient to achieve a similar reduction in MIA PaCa-2-derived microtumors (Figure 6C and 6D). This is in agreement with the poorer response of MDA-MB-231 to Deltaflexin3 observed in 2D proliferation data (Figure 4A). The selected concentrations of 2.5 μM Deltaflexin3 and 30 μM Sildenafil for the combination treatment furthermore fall into a concentration regime that shows high synergism in both the ZIP- and the BLISS/LOEWE-based analysis (Figure 5D). Consistent with the synergistic increase in efficacy observed for the combination of Deltaflexin3 and Sildenafil in BRET and proliferation assays, MIA PaCa-2-derived microtumor growth was more potently reduced by the combination than by each compound alone (Figure 6C and 6D).

## Discussion and Conclusions

Here, we developed Deltaflexin3, a nanomolar active and highly soluble PDE6Di with much improved on-target activity as compared to previous reference inhibitors Deltarasin, Deltazinone1, and Deltasonamide1. We show that combinations of Deltaflexin3 with the approved drug Sildenafil synergistically inhibit intracellular binding of K-Ras to PDE6D and can act favorably or even synergistically on Ras signaling, proliferation, and ex vivo tumor growth of MIA PaCa-2 cells. Other cancer cell lines with a high *PDE6D* and low *PRKG2* dependence may respond similarly, as suggested by data of the SW403 cell line.

Within our dedicated series of 16 compounds, computational docking enabled us to generate several low and subnanomolar binders of PDE6D, which are thus equally potent as previous trailblazer compounds Deltazinone1 and Deltasonamide1. In the first-round compounds, the piperidyl substituent in position R^1^ reduces the affinity most severely, while the ethyl substituent supports high-affinity binding to PDE6D. Building on the best-performing first-round compound Deltaflexin3, the affinity in second-round compounds was increased or at least maintained in all cases, with the exception of **14**. This compound carries the nitro substituent in the ortho position of the benzoic acid moiety, while its meta-substituted derivative **12** exhibits again a low nanomolar affinity. Finally, the methyl-ester derivative of **12**, compound **11**, shows the highest affinity among all tested inhibitors (*K*_D_ = 0.3 ± 0.1 nM).

Surprisingly, we measured lower, only submicromolar, affinities when employing F-Rheb instead of F-Ator as a probe in our fluorescence polarization-based assay. Interestingly, the submicromolar affinities are more in line with the micromolar activities observed in our BRET and proliferation assays (Data S3). Previously, we also measured only low micromolar affinities for first-generation Deltaflexins and Deltarasin using the F-Rheb probe and in an alternative surface plasmon resonance-based assay that detected the disruption of farnesylated K-Ras binding to PDE6D.^27^ Hence, it appears that F-Ator-derived affinities are systematically higher than F-Rheb-derived affinities. The reasons for this are unclear, but it is conceivable that two molecules of F-Ator insert into the hydrophobic pocket of PDE6D, which may be large enough to accommodate also dually geranylgeranylated cargo.^9^ If only one is displaced, the other F-Ator molecule might be able to stabilize the binding of compounds. However, when comparing the F-Rheb-derived affinities from our previous compound Deltaflexin2 (*K*_D_[F-Rheb] = 7.17 μM) and Deltaflexin3 (*K*_D_[F-Rheb] = 0.63 μM), a more than 10-fold improvement in affinity becomes apparent.

Another important aspect of our PDE6Di development effort is the dedicated off-target analysis, which has not been done previously. Of note, all of our first-round compounds display less off-target effects in both assays than Deltazinone1, the reference compound with the least off-target activity (Figure 3). From our BRET-based off-target analysis, it appears that compounds with a PDE6D affinity below ∼3 nM are more likely to engage UNC119A as an off target (Figure 3B–D). It is plausible that also related UNC119B would be engaged in this way.^43^ Depending on the expression levels of such lipid-binding proteins, they may effectively act as sinks for PDE6Di. It is plausible to assume that raising compounds with a higher affinity to a highly hydrophobic pocket will render them likewise more hydrophobic. It is possible that this trend then also increases the likelihood of binding to other hydrophobic pockets, such as that of UNC119A.

Importantly, the highest K-RasG12V selectivity is seen for Deltaflexin3 (Figure 2B), consistent with its lowest off-target effect in both the BRET-based assay looking at UNC119A engagement and its assessment in *PDE6D* KO MEFs (Figure 3). Overall, K-RasG12V-BRET selectivity (Figure 2B) and PDE6D selectivity derived from cell proliferation data of WT and KO-MEFs (Figure 3A) show a strong correlation for our compounds (Figure S3B), supporting that our assessment selects for least off-target activity.

PDE6Di development could in the future adopt strategies illustrated in nature. When looking at known cargos of PDE6D, it becomes apparent that their affinity is not modulated within the hydrophobic pocket but outside of it, at its entry site.^8,9,12^ Contacts with entry site residues are typically not exploited with PDE6Di, albeit our second round of compounds was extended with this goal in mind. However, docking data suggest that the flexible hexamethylene linker in **15** was disadvantageous in this regard, as it curled up in the pocket (Figure 1E).

Notably, for monoprenylated cargo, it is known that the four residues upstream of the prenylated cysteine significantly modulate the cargo affinity to PDE6D.^8^ While K-Ras has only a moderate micromolar PDE6D affinity (*K*_D_ = 2.3 μM^8^), the INPP5E-derived peptide has a high, nanomolar affinity (*K*_D_ = 3.7 ± 0.2 nM^12^), and this solely depends on two amino acids in the four-residue stretch upstream of the farnesylated cysteine.^9^

The potential of this kind of affinity modulation is essentially illustrated by our Sildenafil data (Figure 5A), as Ser181 of K-Ras is part of that four-residue stretch next to the farnesylated cysteine. Therefore, future PDE6Di may rather target that region of the protein while using a minimal hydrophobic stretch to anchor inside the hydrophobic pocket. We propose that “plugging” rather than “stuffing” the hydrophobic pocket of PDE6D with novel inhibitors may present a way forward.

Inhibitors of Ras membrane anchorage are expected to shut down Ras-signaling output.^5^ For instance, farnesyl-transferase inhibitors that block the enzyme-mediating Ras farnesylation are now applied with some success in *HRAS* mutant head and neck cancers.^44^ While some PDE6Di were shown to dislodge K-Ras more or less from the plasma membrane within 60–90 min,^7,15,16,26^ only in some cases was evidence for a moderate effect on Ras signaling provided.^16,24,26^ Nevertheless, all of these PDE6Di demonstrated cell killing activity in *KRAS* mutant pancreatic or colorectal cancer cells; however, these are assays that cannot detect off-target activities.

One explanation for these discrepancies could be that only a fraction of K-Ras that is trafficked to the plasma membrane does actually depend on PDE6D. We therefore compared the knockdown of *PDE6D* or that of the alpha subunit of farnesyl- and geranylgeranyl-transferases (*FNTA*) with Mevastatin treatment, which would completely block K-Ras membrane anchorage, using our BRET assay that detects functional K-RasG12V membrane organization (Figure S1C–F). These data show that knockdown of *FNTA* is 49% as effective as Mevastatin treatment, while PDE6D knockdown is only 26% as efficient (Figure S1C). This suggests that only between one-quarter and one-half of functional K-Ras membrane anchorage depends on PDE6D. It is plausible to assume that other trafficking chaperones compensate and salvage K-Ras membrane anchorage, thus buffering the loss of PDE6D activity.

It may therefore not be astonishing that both reference PDE6Di Deltazinone1 and our own compounds have a small effect on the activation, in particular, of the MAPK-pathway but also on the AKT/mTORC1-pathway (Figure 4C and 4D and Figure 6A and 6B). However, when combined with Sildenafil, a significant reduction of activity in both pathways is observed (Figure 6A and 6B). Indeed, this combination may in general be a way forward for PDE6Di application as it focuses the inhibitory activity on K-Ras. Apart from K-Ras, only 15 other small GTPases can potentially be modulated in their binding to PDE6D by both PDE6Di and Sildenafil (Data S4).

This drug combination also showed promise for the antitumorigenic activity of our most selective PDE6Di, Deltaflexin3 (Figure 6C and 6D). However, not all *KRAS* mutant cancer cell lines respond synergistically to the Deltaflexin3/Sildenafil combination (Figure 5C and 5D). MIA PaCa-2 and SW403 may be more responsive in this regard as they have a genetic dependence on both *KRAS* and *PDE6D* while being not dependent on *PRKG2* (the gene of PKG2) (Figure S3D). Thus, inhibition of PDE6D and activation of PKG2 are expected to be particularly efficacious in these two cell lines.

One would therefore expect that this combination could find its application in the treatment of a subset of *KRAS* mutant cancers that more often have a high *PDE6D* and a low *PRKG2* expression level, such as colorectal cancer (Figure S4C). However, our analysis of the overall survival of patients with this expression signature across *KRAS* mutant cancers in the PanCanAtlas data set shows that they have a significantly better survival than those with the opposite signature (low *PDE6D*/high *PRKG2*) (Figure S4D). This may indicate a protective effect of the high-*PDE6D*/low-*PRKG2* signature that should not be drug targeted by a PDE6Di/Sildenafil combination.

This begs the question as to what specific role PDE6D has for K-Ras trafficking. Given that PDE6D is a major trafficking chaperone of ciliary cargo and that a K-Ras mutant has indeed been observed inside the primary cilium,^9^ it is possible that PDE6D inhibition also affects trafficking of K-Ras to this destination. However, the significance of such an inhibition in ciliated cells is unclear given that no function of K-Ras in the cilium is known. Besides, cancer cells are typically not ciliated,^45^ and it would thus not be clear what effect PDE6D inhibition could have in this context.

Another complication of PDE6D as a drug target is its intrinsically broad cargo spectrum.^8,9^ Therefore, its inhibition will affect not only K-Ras and thus *KRAS* mutant cancer cells but also a host of PDE6D cargos. Finally, the ontogenetic role of *PDE6D* may be worth considering. Loss of function mutations of *PDE6D* during development lead to the multisystemic ciliopathy Joubert Syndrome.^46^ The deletion of *PDE6D* in mice does not cause gross developmental abnormalities as mice are fertile and viable.^47^ Some progressive defects in photoreceptor physiology were however observed as well as an overall reduced body weight. Even though such genetic data do not exactly translate into the effects observed with inhibitors that are typically applied to aged cancer patients, more insight into the PDE6D biology in conjunction with K-Ras seems warranted.

In conclusion, we provide data that support a novel conceptual framework for the future development and application of PDE6Di to be redesigned as “plugs” and to be used in combination with PKG2 activators, such as approved Sildenafil. However, we also recommend improving our understanding of the PDE6D involvement in cancer and the consequences of drug targeting it. With our novel, potent PDE6D inhibitor Deltaflexin3, which has the highest K-Ras selectivity and lowest off-target activity so far described, we are now providing the currently best tool compound to investigate and further validate the significance of PDE6D (patho)biology.

## Materials and Methods

### Materials and Equipment

All materials, reagents, and equipment are listed in Table S1.

### Compound Synthesis and Analysis

A comprehensive description of the compound synthesis and analysis by NMR and MS can be found as Data S2. All compounds are >95% pure by HPLC analysis.

### Cell Lines

HEK293-EBNA (HEK) cells were a gift of Florian M. Wurm, EPFL, Lausanne, Switzerland, and were cultured in Dulbecco’s modified Eagle’s medium (DMEM, #41965-039). WT MEF and MEF *PDE6D* KO cells (obtained from Richard A. Kahn, Emory University School of Medicine, Atlanta, GA, USA) were cultured in DMEM. NCI-H358, MDA-MB-231, and IGR-39 were maintained in Roswell Park Memorial Institute medium (RPMI, #52400-025). PANC-1, MIA PaCa-2, Hs 578T, and T24 were maintained in DMEM. SW620, SW480, and SW403 were maintained in Leibovitz’s L-15 medium (#11415-064). All media were supplemented with 10% v/v fetal bovine serum (#10270-106), 2 mM l-glutamine (#25030-024), and penicillin 100 U/mL/streptomycin 100 μg/mL (#15140-122) (complete medium). All cell culture media and reagents were from Gibco, Thermo Fisher Scientific. Cells were grown at 37 °C in a water-saturated, 5% CO_2_ atmosphere and subcultured twice a week. Cell lines SW620, SW403, and SW480 were cultured without CO_2_.

### Bacterial Strains

Competent *E. coli* DH10B and *E. coli* BL21 Star (DE3)pLysS were grown in Luria–Bertani (LB) medium at 37 °C with appropriate antibiotics unless otherwise mentioned.

### Expression Constructs

All expression constructs were produced by multisite Gateway cloning technology as described.^48^ Briefly, entry clones had compatible LR recombination sites, encoding the CMV promoter, Rluc8, or GFP2 tag and a gene of interest. The location of the tag in the expression constructs is indicated by its position in the construct name, i.e., a tag at the N-terminus of the protein of interest is written before the name of the protein. Plasmids with cDNAs were obtained either from the Ras-Initiative (K-Ras4BG12V and H-RasG12V, both from the RAS mutant clone collection, kit #1000000089 and PDE6D #R702-E30) or by custom synthesis from GeneCust (human c-Src kinase and human UNC119A inserted in pDONR221). The three entry clones of promoter, tag, and gene of interest were then inserted into pDest-305 or pDest-312 as a destination vector using Gateway LR Clonase II enzyme mix (#11791020, Thermo Fisher Scientific). The reaction mix was transformed into ccdB-sensitive *E. coli* strain DH10B, and positive clones were selected in the presence of ampicillin. The His6-MBP-Tev-PDE6D construct for PDE6D protein production was obtained from the Ras-Initiative (#R702-X31-566).

### In Silico Docking of Compounds

The synthetic rationale for first-round compounds was based on computational docking. Three-dimensional coordinates for the molecular structure and sequence of the open and closed conformations of the PDE6D protein (PDB ID 4JV8 and 1KSH, respectively) were retrieved from the RCSB Protein Data Bank.^7^ The 3D structures of all docked compounds were constructed using Maestro software in the Schrödinger software (Schrödinger Release 2019-2; Maestro, Schrödinger, LLC, New York, NY, USA, 2019). The geometry optimization of docked compounds was performed using the OPLS3 force field.^49^ The Powell conjugated gradient algorithm method was applied with a convergence criterion of 0.01 kcal/(mol Å) and maximum iterations of 1000.

Molecular docking simulations were performed by using the program Glide.^29^ Flexible compound, extra precision mode, and the Epik state penalties were included in the protocol. The MM-GBSA method with the VSGB 2.0 solvation model was used to calculate compound binding affinities.^50^ For MM-GBSA calculations, residues within a distance of 8.0 Å from the compound were assigned as flexible.

Computational evaluations to derive second-round compounds were slightly different. While using the same protein data as for first-round compounds, the putative binding pocket of PDE6D was reinferred using the software SeeSAR v10.3 (“SeeSAR” 2020) with default parameters and prior domain knowledge to select and refine the most relevant pocket. Compound chemical formulas, defined as SMILES strings, were converted to 3D structures using OpenBabel v2.3.2 with default parameters.^51^ Compounds were docked to PDE6D (PDB ID 4JV8) using SeeSAR v10.3, and the optimal docking pose was manually selected by ranking poses according to their predicted binding affinity and filtering compounds to ensure acceptable lipophilic compound efficiency, limited torsions of the compound backbone, and minimal intra- and intermolecular clashes of the resulting protein–ligand complex.

### Expression and Purification of PDE6D

Recombinant PDE6D protein was produced according to a published protocol that was adapted.^8^ Briefly, *E. coli* BL21 Star (DE3)pLysS strain was transformed with pDest-His6-MBP-Tev-PDE6D and grown at 37 °C in LB medium supplemented with ampicillin at 1:1000 dilution from 100 mg/mL stock. When OD reached 0.6, protein expression was induced by adding isopropyl β-d-1-thiogalactopyranoside (IPTG, #437145X, VWR) at 16 °C overnight. Next, the 4 L cultures were pelleted by centrifugation; the pellets were rinsed with PBS and stored at −20 °C until purification.

Purification was conducted using an ÄKTA pure chromatography system (Cytiva). All buffers were degassed by placing them for 5 min in an ultrasonic bath. The cells were lysed by sonication on ice in a buffer composed of 50 mM Tris-HCl, pH 7.5, 150 mM NaCl, 1 mM β-mercaptoethanol, 0.5 mg/mL lysozyme (#89833, Thermo Fisher Scientific), and protease inhibitor cocktail (#A32955, Pierce). For sonication, a Bioblock Scientific ultrasonic processor instrument (Elmasonic S 40 H, Elma) was used. Lysates were cleared by centrifugation at 18 000 g for 20 min at 4 °C. Cleared supernatant was loaded onto a prepacked HisTrapHP column (#17-5248-02, Cytiva) equilibrated in a binding buffer, which had the same composition as lysis buffer but without lysozyme and containing 35 mM imidazole. After washing with 20 column volumes, the bound material was eluted by isocratic elution using 100% of eluting buffer (50 mM Tris-HCl, pH 7.5, 150 mM NaCl, 1 mM β-mercaptoethanol, 500 mM imidazole). The eluted fractions were analyzed by resolving on 4–20% SDS-PAGE (#4561094 or #4651093 BioRAD) and stained with Roti-Blue quick (#4829-2, Carl ROTH). Fractions were concentrated on AmiconUltra centrifugal filters (molecular weight cutoff, MWCO, of 30 kDa, Merck Millipore) by centrifuging at 7500*g* and pulled for dialysis into buffer containing 50 mM Tris-HCl, pH 7.5, 150 mM NaCl, and 3 mM DTE using a D-Tube dialyzer with molecular weight cutoff (MWCO) of 12–14 kDa, overnight at 4 °C. Next, samples were centrifuged for 15 min at 4000*g* and 4 °C and then loaded onto a size exclusion chromatography column (HiLoad 16/600 Superdex 75 pg, with a 120 mL column volume, #28989333, Cytiva) at a flow rate of 1 mL/min with elution with two column volumes. Fractions were analyzed as above and then concentrated to a volume of about 500 μL. In the next step, protein tags were removed by tobacco etch virus (TEV) protease (#T4455, Sigma-Aldrich) (1:25 w/w, TEV/fusion protein) during overnight dialysis. This step was repeated twice, with 50% and 70% approximate cleavage efficiencies. The cleaved mixture was loaded onto a HisTrapHP column, and the nonbound (tag-free) PDE6D was collected. The collected PDE6D fractions were concentrated using MICROSEP Advance (MWCO 10 kDa, # 88527, Pierce) by centrifugation at 7500*g* and 4 °C. The sample was finally dialyzed overnight in a buffer composed of 20 mM HEPES, pH 7.4, 150 mM NaCl, 5 mM MgCl_2_, and 1 mM TCEP. The PDE6D final concentration of 245.3 μM was determined by Bradford assay. The final purification yield from 4 L of starting bacterial culture was 890 μg of PDE6D.

### Fluorescence Polarization Assay

The IC_50_ and *K*_D_ of compounds to purified PDE6D were determined in a displacement assay using fluorescein-labeled Atorvastatin (F-Ator) or fluorescein-labeled farnesylated Rheb (F-Rheb) peptide as probes.^7,14^ F-Ator was used at a 5 nM concentration with 5 nM of PDE6D, and F-Rheb peptide was used at a 0.5 μM concentration with 2 μM PDED. Assays were carried out in black low-volume round-bottom 384-well plates (#4514, Corning) with a reaction volume of 20 μL for F-Ator- and 10 μL for F-Rheb-based experiments. Compounds were 3-fold diluted in assay buffer (DPBS no Ca^2+^/Mg^2+^; #14190-094, Gibco) with 0.05% CHAPS (#1479, Carl Roth) for F-Ator-based experiments or in a freshly prepared buffer composed of 30 mM Tris, 150 mM NaCl, and 3 mM dithiothreitol for F-Rheb-based experiments, as described previously.^27,52^ The fluorescence polarization signals were read on the CLARIOstar plate reader (BMG Labtech GmbH) with λ_ex_ = 482 ± 8 nm and λ_em_ = 530 ± 20 nm at 25 °C. The blank-corrected milli polarization value (mP or P × 1000) calculated from the MARS (BMG Labtech) program was plotted against the logarithmic concentration of inhibitors. The data were fitted into the log inhibitor vs response 4-parametric equation of Prism (GraphPad) to obtain the IC_50_ values. The IC_50_ values were converted into *K*_D_ using the modified Cheng–Prusoff equation, , where *K*_D_ is the dissociation constant between PDE6D and inhibitor, [L] is the ligand or fluorescent probe concentration used in the assay, and *K*_d_ is the dissociation constant between the PDE6D and the ligand or fluorescent probe.^27^ The reported *K*_d_ values were 7.1 ± 4 nM for F-Ator to PDE6D^7^ and from 0.15^14^ to 0.45 μM^12^ for F-Rheb to PDE6D. The mean of the F-Rheb *K*_d_ value of 0.3 μM was used for the calculations. Note that the concentration of PDE6D is not part of the equation.

### Kinetic Solubility Assay

For each compound to be tested, a 50 mM DMSO stock solution was used to prepare a standard linearity stock solution, the solvent being acetonitrile. From this standard linearity stock solution, standard linearity sample solutions at 5 different concentrations (0.01, 0.02, 0.05, 0.10, and 0.20 mg/mL) were prepared in acetonitrile and tested by HPLC to obtain the corresponding peak areas. With the concentration as the horizontal coordinate (*x*) and the peak area as the vertical coordinate (*y*), a linear curve was drawn to obtain the linear equation *y* = *ax* + *b*, which serves as the basis for quantifying solution concentrations. In order to determine the kinetic solubility, a determined volume of DMSO stock solution was given into a tube, and then a determined volume of PBS, pH 7.4, was added until formation of a precipitate. These sample solutions were then shaken on the oscillator at 37 °C for 0, 4, or 24 h. Following centrifugation, the supernatant was filtered into an HPLC injection vial, and the peak area of each filtered sample solution was obtained by HPLC. The kinetic solubility of each compound at 37 °C in PBS, pH 7.4 after shock times of 0, 4, or 24 h was then calculated using the linear equation *y* = *ax* + *b* established before.

### Bioluminescence Resonance Energy Transfer (BRET) Assay

BRET assays were essentially performed as described by us previously.^36,53,54^ Briefly, 150 000–200 000 HEK293-EBNA cells were plated in 1 mL of complete DMEM per well of 12-well cell culture plates (#665180, Greiner bio-one, Merck KGaA). After 24 h, donor and acceptor plasmids were transfected into cells using 3 μL of jetPRIME transfection reagent (#114-75, Polyplus) following the manufacturer’s instructions.

For BRET donor saturation titration experiments, the concentration of donor plasmid (50 ng) was kept constant and the concentration of acceptor plasmid was increased from 0 to 1000 ng. The empty pcDNA3.1 plasmid was used to top up the total DNA load per well to 1050 ng.

After determination of the optimal acceptor to donor plasmid ratio from the titration experiments (A/D plasmid ratio 20:1 for GFP2-K-RasG12V/Rluc8-PDE6D, 5:1 for GFP2-K-RasG12V/Rluc8-K-RasG12V, 3:1 for GFP2-HRasG12V/Rluc8-HRasG12V, and 20:1 for Src-GFP2/UNC119A-Rluc8), compound dose–response experiments were performed. Twenty-four hours after transfection, cells were treated for another 24 h with DMSO 0.1% v/v as vehicle control or with compounds at 5–8 different concentrations ranging from 20 to 0.15 μM, prepared as 2-fold dilution series in complete medium.

To study the effect of siRNA-mediated knockdown, cells were plated and after 24 h cotransfected with 50 nM siRNA and 500 ng of plasmid DNA per well (same A/D plasmid ratio as described above) using 4 μL of Lipofectamine 2000 (#11668019, Thermo Fisher Scientific) in Opti-MEM medium (#31985062, Gibco).

BRET measurements were performed on a CLARIOstar plate reader at 25 °C after 48 h as described.^36,53,54^ Technical quadruplicates were measured using specific channels for the luminophores (GFP2-acceptor signal, RFU, at λ_ex_ = 405 ± 10 nm and at λ_em_ = 515 ± 10 nm; after 10 μM coelenterazine 400a (#C-320, Gold Biotechnology) addition, simultaneous recording of Rluc8 signal as donor signal, RLU, λ_em_ = 410 ± 40 nm and for the BRET signal at λ = 515 ± 15 nm). The BRET ratio was calculated as before.^36,53,54^

For BRET donor saturation titration experiments, the BRET ratio was plotted against the relative expression. The relative expression of acceptor to donor ([acceptor]/[donor]) was determined as the ratio between RFU and RLU. All independent repeat experiments were plotted at once using these normalized data, i.e., BRET ratio against relative expression. The data were fitted into one phase association equation of Prism 9 (GraphPad), and the top asymptote *Y*_max_ value was taken as BRET_top_. It represents the maximal BRET ratio reached within a defined [acceptor]/[donor] ratio range. Statistical analysis between the BRET_top_ values was performed using student’s *t* test.

### 2D Cell Proliferation Assay

Cancer cells were seeded at a density of 1000 cells/100 μL of complete medium into 96-well cell culture plates (#655180, Greiner bio-one, Merck KGaA). After 24 h, control and test compounds were added to the cells with DMSO (0.1% v/v) as a vehicle control. Compound activities were analyzed from 9-point dose–response curves with compounds prepared as 2-fold dilution series ranging from 40 to 0.15 μM (PDE6Di and FTI-277) or from 20 to 0.02 μM for MAPK-control compounds. Following incubation for 72 h with the compounds, the cell viability was assessed using the alamarBlue reagent (#DAL1100, Thermo Fisher Scientific) according to the manufacturer’s instructions. After addition of alamarBlue reagent at a 10% v/v final volume, cells were incubated for 2–4 h at 37 °C. Then, the fluorescence intensity was read at λ_ex_ = 530 ± 10 nm and λ_em_ = 590 ± 10 nm at 25 °C using a CLARIOstar plate reader. The obtained raw fluorescence intensity data were normalized to vehicle control (100% viability) and plotted against the compound concentration.

### Drug Sensitivity Score Analysis (DSS3)

As described before,^53^ a drug sensitivity score (DSS) analysis was performed in order to quantify the drug sensitivity with a more robust parameter than the IC_50_ or EC_50_ values. DSS values are normalized area under the curve (AUC) measures of dose–response inhibition data, where the DSS3 score values drug responses more that are achieved across a broad concentration range.^30^ Drug response data from BRET assays or 2D cell proliferation assays were prepared according to the example file on the Breeze Web site (https://breeze.fimm.fi/), uploaded, and analyzed.^55^ The output file included DSS3 scores as well as several other drug sensitivity measures such as EC_50_ and AUC.

### Synergy Analysis of Drug Combinations

The synergistic potential of compounds was analyzed essentially as described before.^54^ For PDE6D/K-RasG12V BRET experiments, full dose–response analyses of Deltaflexin3 (between 10 and 0.014 μM) or Sildenafil (between 320 and 0.018 μM) alone or for Deltaflexin3 in combination with Sildenafil maintained at a fixed concentration of 10, 20, or 30 μM were performed. For 2D proliferation experiments, full dose–response analyses of Deltaflexin3 (between 80 and 0.156 μM) or Sildenafil (between 160 and 0.312 μM) alone or for Deltaflexin3 in combination with Sildenafil maintained at a fixed concentration of either 10, 20, 30, or for some cell lines 40 μM were performed. Comparison between the drug response profiles of the combinations and the profiles of each single agent was then carried out using the web application SynergyFinder (Version 3.0; https://synergyfinder.fimm.fi).^40^ We employed the zero interaction potency (ZIP) model, which assumes that the potencies of individual drugs are not influenced by each other. The detailed higher order formulations to quantify the synergy were defined in ref (56). To enhance accuracy in identifying true synergy, we implemented the BLISS/LOEWE consensus synergy score. This method brings together various synergy reference models, including Bliss, Loewe, and HSA, that are applicable to both pairwise and higher order combination data sets, effectively minimizing false positive results.^40^

### ATARiS Gene Dependence Score

Gene dependence scores of selected genes of interest for cancer cell lines used in this study were obtained from the drive data portal (https://oncologynibr.shinyapps.io/drive/). The DRIVE project has provided the dependence data of 7837 genes for 398 cancer cell lines, as determined by large-scale RNAi screening in cell viability assays.^39^ A double-gradient heatmap for the extracted gene dependence scores was then generated using GraphPad Prism software.

### Immunoblotting

Following a 16 h serum starvation, MIA PaCa-2 cells were treated with 0.1% v/v DMSO vehicle control or with compounds at 37 °C for 4 h and then stimulated with 200 ng/mL human epidermal growth factor (hEGF, #E9644, Sigma) at 37 °C for 10 min. In situ cell lysis was performed in ice-cold lysis buffer (50 mM Tris-HCl pH 7.5, 150 mM NaCl, 0.1% v/v SDS, 5 mM EDTA, 1% v/v Nonidet P-40, 1% v/v Triton X-100, 1% v/v sodium deoxycholate, 1 mM Na_3_VO_4_, 10 mM NaF, 100 μM leupeptin, and 100 μM E64D protease inhibitor) supplemented with a cocktail of protease inhibitors (#A32955, Pierce) and a cocktail of phosphatase inhibitors (PhosSTOP, #4906845001, Roche Diagnostics GmbH). After lysate clarification, the total protein concentration was determined by Bradford assay using the Quick Start Bradford 1× Dye reagent (#5000205, Bio-Rad) and BSA (#23209, Thermo Fisher Scientific) as a standard. Proteins (50 μg per lane) were resolved by SDS-PAGE in a 10% v/v homemade polyacrylamide gel under reducing conditions and transferred to a nitrocellulose membrane by a semidry transfer (kit #1704272, Bio-Rad). Membranes were saturated in phosphate-buffered saline (PBS) containing 2% w/v bovine serum albumin (#A6588, AppliChem GmbH) and 0.2% Tween for 1 h at room temperature and then incubated with primary antibodies overnight at 4 °C. For phospho-ERK detection, a combination of mouse antiphospho-ERK and rabbit anti-ERK was used. For phospho-S6, phospho-MEK1/2, or phospho-Akt (Ser473) detection, combinations of rabbit antiphospho-protein and mouse antiprotein antibodies were used (see Table S1). Incubation with secondary antibodies was performed for 1 h at room temperature. Each antibody incubation was followed by at least three wash steps in PBS supplemented with 0.2% v/v Tween 20. Signal intensities were quantified using the Odyssey Infrared Image System (LI-COR Biosciences). The ratio between the intensities obtained for phosphorylated protein versus total protein was calculated and then normalized to the sum of all of the ratios calculated for one blot to make blots comparable by accounting for technical day-to-day variability. For representative purposes, data were scaled to the controls present on each blot and are represented as the mean ± SEM of at least three independent biological repeats. The slope of the dose–response data was determined from fitting a line using GraphPad Prism. For each blot, either β-actin or GAPDH levels were determined as a loading control.

### Chorioallantoic Membrane (CAM) Assay

Fertilized chicken eggs were obtained from VALO BioMedia GmbH (Osterholz-Scharmbeck, Germany), and on day 1, development of the embryos was started by incubating the eggs at 37 °C in a >60% humidified egg hatcher incubator (MG200/300, Fiem). A small hole was made with the help of an 18 Gauge needle (#305196, Becton Dickinson) into the narrower end of each egg on day 3 and was kept covered with parafilm to avoid contamination. On day 8, 2 × 10^6^ MDA-MB-231 cells or 3.5 × 10^6^ MIA PaCa-2 cells were resuspended in 10 μL of cell culture medium without FBS and mixed 1:1 with Matrigel (#356234, Corning). This mix was then deposited in sterilized 5 mm diameter plastic rings cut from PCR tubes (#683201, Greiner bio-one, Merck KGaA) on the surface of a chicken embryo chorioallantoic membrane. After 1 day, the growing tumors were treated with a volume identical with the deposited cell suspension of 0.2% v/v vehicle control or test compounds 2× concentrated in medium without FBS.^32,41^ Treatment was performed daily, and after 5 days of treatment, the microtumors were harvested at day 14 of embryo development. Then, the tumor weight was determined using a balance (E12140, Ohaus).

### Tumor Xenograft Experiments in Mice

Tumor xenograft studies were performed at the Turku Center for Disease Modeling (Turku, Finland). The animal studies were authorized by the National Animal Experiment Board of Finland and were performed according to the guidelines of the Institutional Animal Care and Use Committees of the University of Turku. Briefly, mycoplasma- and mouse pathogen-free MDA-MB-231 cells were suspended in serum-free culture medium (25 × 10^6^ cells/mL) and then mixed (1:1) with Matrigel (Corning Matrigel Basement Membrane Matrix, cat. # 354234). Female athymic nude mice (Hsd:Athymic Nude-Foxn1) were inoculated subcutaneously into one flank with 2.5 × 10^6^ MDA-MB-231 cells/mouse. After 5 days, mice were allocated to two groups, MDA-MB-231 control (*n* = 10) and MDA-MB-231 Deltaflexin3 treated (*n* = 10), based on body weight and tumor volume, following an optimized group design protocol.^57^ One week after inoculation, treatment was started. The compound Deltaflexin3 was diluted in NaCl 0.9% vehicle solution, and 10 mg/kg in 200 μL was injected daily intraperitoneally. The tumor volume was monitored three times a week by caliper measurement, and animals were weighed once a week. The volume of the tumors was calculated according to the following formula: *W*^2^ × *L*/2 (*W* = shorter diameter, *L* = longer diameter of the tumor). In total, tumors were grown for 3 weeks.

### Survival Analysis

All data were retrieved from TCGA Pan-Cancer Atlas (https://dev.xenabrowser.net/heatmap/) (PANCAN). The 647 cancer samples with nonsilent somatic *KRAS* mutation as defined in Xena were selected (https://ucsc-xena.gitbook.io/project/overview-of-features/visual-spreadsheet/mutation-columns). Expression data was retrieved for *PDE6D* and *PRKG2* genes data in “batch effects normalized mRNA data” units, and samples were split in 4 groups according to high or low expression of each gene, setting the limit at median expression value for each gene. The difference between the two curves was tested using Kaplan Meyer estimation. Data analyses were performed in R version 4.2.1.^58^ Survival analyses and plots were done using survival v.3.4^59^ and survminer v 0.4^60^ libraries.

### Quantification and Statistical Analysis

For statistical analysis and plot preparation, GraphPad Prism (version 9.5.1 for Windows, GraphPad Software, USA, www.graphpad.com) was used. The sample size *n* represents the number of independent biological repeats and is indicated in the respective figure legends. All graphs show mean values ± SEM across all technical and biological repeats. We determined statistical differences by employing one-way ANOVA with Tukey’s multiple comparison test unless otherwise mentioned in the legends. A *p* value of <0.05 is considered statistically significant. Statistical significance levels are annotated in the plots as * = *p* < 0.05; ** = *p* < 0.01; *** = *p* < 0.001; **** = *p* < 0.0001.

## Data Availability

This study did not report standardized datatypes. All unique/stable reagents generated in this study are available from the corresponding author with a completed materials transfer agreement.

## Supporting Information Available

Data supplementing Figure 2; confocal microscopy-based analysis of K-RasG12V plasma membrane localization after treatment, supplementing Figure 2; data supplementing Figures 2–5; data supplementing Figure 6; materials and equipment used in this study (PDF)
Overview of the chemical structures of the compounds used to generate hybrid compounds for first-round in silico screening and hybrid compound overview; compound synthesis and analysis (PDF)
Data 3: SMILES and activity data of all investigated compounds; survey of established and predicted PDE6D cargo GTPases (XLSX)
Data 4: Activity summary report of tested PDE6D inhibitors (CSV)

## Abbreviations Used

AKT: protein kinase B
Arl: ADP ribosylation factor-like GTPase
Ator: Atorvastatin
BRET: bioluminescence resonance energy transfer
CAM: chorioallantoic membrane
DSS: drug sensitivity score
ERK: extracellular signal-regulated kinase
FITC: fluorescein isothiocyanate
FNTA: gene of FTase/GGTase I alpha subunit
FTase: farnesyl transferase
GAPDH: glyceraldehyde-3-phosphate dehydrogenase
GGTase I: geranylgeranyl transferase I
HVR: hypervariable region
INPP5E: inositol polyphosphate-5 phosphatase E
MBP: myelin basic protein
MDCK: Madin–Darby canine kidney
MEF: mouse embryonic fibroblast
MEK: MAPK/ERK kinase
MM-GBSA: molecular mechanics/generalized Born surface area
PDE5: phosphodiesterase 5
PDE6D: phosphodiesterase 6 delta
PKG2: cGMP-dependent protein kinase 2
PM: plasma membrane
PROTAC: proteolysis-targeting chimera
Rab: Ras-related protein
Rac: Ras-related C3 botulinum toxin substrate
RFU: relative fluorescence unit
Rheb: Ras homologue enriched in brain
Rho: Ras homologue
RLU: relative luminescence unit
Rluc: Renilla luciferase
S6: ribosomal protein S6
Src: Src proto-oncogene nonreceptor tyrosine kinase
UNC119: Uncoordinated 119
