# Supplementary material for: An Improved PDE6D Inhibitor Combines with Sildenafil To Inhibit KRAS Mutant Cancer Cell Growth
Source: J Med Chem. 2024 May 17;67(11):8569–84. doi: 10.1021/acs.jmedchem.3c02129 (PMC11181323; doi:10.1021/acs.jmedchem.3c02129)
Supplement: Supplementary file 1 — jm3c02129_si_001.pdf [file jm3c02129_si_001.pdf]

Supporting Information

An improved PDE6D inhibitor combines with Sildenafil to inhibit *KRAS* mutant cancer cell growth

Pelin Kaya [1], Elisabeth Schaffner-Reckinger [1], Ganesh babu Manoharan [1], Vladimir Vukic [2], Alexandros Kiriazis [3,#], Mirko Ledda [4], María Burgos Renedo [1], Karolina Pavic [1], Anthoula Gaigneaux [5], Enrico Glaab [4], Daniel Kwaku Abankwa [1,3 *]

[1] Cancer Cell Biology and Drug Discovery Group, Department of Life Sciences and Medicine, University of Luxembourg, 4365 Esch-sur-Alzette, Luxembourg

[2] Faculty of Technology, University of Novi Sad, 21000 Novi Sad, Serbia

[3] Turku Bioscience Centre, University of Turku and Åbo Akademi University, 20520 Turku, Finland

[4] Luxembourg Center for Systems Biomedicine, University of Luxembourg, 4365 Esch-sur-Alzette, Luxembourg

[5] Bioinformatics Core, Department of Life Sciences and Medicine, University of Luxembourg, 4365 Esch-sur-Alzette, Luxembourg

[#] current address: Orion Corporation, Orion Pharma, Espoo, Finland

[*] Corresponding author: daniel.abankwa@uni.lu

Contents of Supporting Information

This PDF File containing supplementary Figures and Table S1:

Figure S1: Data supplementing main **Figure 2**.

Figure S2: Confocal microscopy-based analysis of K-RasG12V plasma membrane localization after treatment, supplementing main **Figure 2**.

Figure S3: Data supplementing main **Figures 2 - 5**.

Figure S4: Data supplementing main **Figure 6**.

Table S1: Materials and Equipment used in this study.

Separate data files:

Data S1 and S2: PDF file. Data S1: Overview of the chemical structures of the compounds used to generate hybrid compounds for first-round in silico screening and hybrid compound overview. Data S2: Compound synthesis and analysis.

Data S3 and S4: Excel File. Data S3 (tabs 1-18): SMILES and activity data of all investigated compounds. Data S4 (tab 19): Survey of established and predicted PDE6D cargo GTPases.

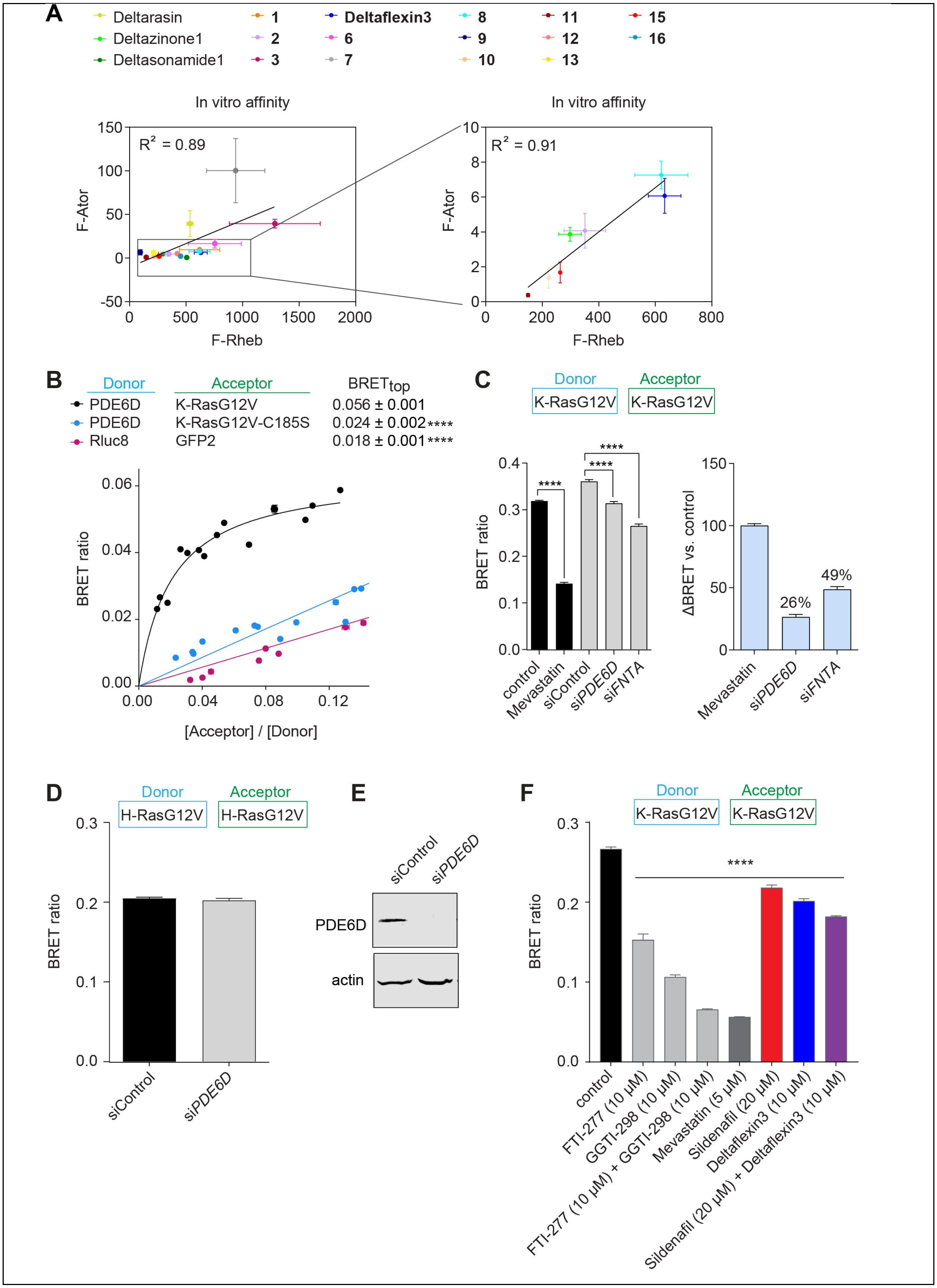
A
Deltarasin
Deltazinone1
Deltasonamide1
1
2
3
Deltaflexin3
6
7
8
9
10
11
12
13
15
16
In vitro affinity
R² = 0.89
F-Ator
F-Rheb
R² = 0.91
B
Donor
Acceptor
BRETtop
PDE6D K-RasG12V 0.056 ± 0.001
PDE6D K-RasG12V-C185S 0.024 ± 0.002****
Rluc8 GFP2 0.018 ± 0.001****
BRET ratio
[Acceptor] / [Donor]
C
Donor
Acceptor
K-RasG12V
K-RasG12V
BRET ratio
ΔBRET vs. control
control
Mevastatin
siControl
siPDE6D
siFNTA
26%
49%
D
Donor
Acceptor
H-RasG12V
H-RasG12V
siControl
siPDE6D
E
siControl
siPDE6D
PDE6D
actin
F
Donor
Acceptor
K-RasG12V
K-RasG12V
control
FTI-277 (10 µM)
GGTI-298 (10 µM)
FTI-277 (10 µM) + GGTI-298 (10 µM)
Mevastatin (5 µM)
Sildenafil (20 µM)
Deltaflexin3 (10 µM)
Sildenafil (20 µM) + Deltaflexin3 (10 µM)

Figure S1. Data supplementing main Figure 2.
(**A**) Correlation plots of PDE6D inhibitor/ PDE6D-K_D values acquired with F-Rheb vs. F-Ator as probes (**Data S3**). Data of compounds **5** and **14** were excluded, due to their much lower affinity.
(**B**) BRET-titration curves of the PDE6D/ K-RasG12V, PDE6D/ K-RasG12V-C185S complexes and only the biosensor tag pair Rluc8/ GFP2 without fused protein-of-interest as controls. The C185S mutation prevents prenylation; $n \geq 3$. For the latter two constructs line fits were done and the BRET-value at the highest [Acceptor]/ [Donor] ratio (mean ± SEM of last two datapoint repeats) was used as BRETtop. Statistical comparisons of BRETtop values to PDE6D/ K-RasG12V were done using two-tailed Student's t-test.
(**C**) K-RasG12V-membrane anchorage evaluated by BRET following treatment with 5 µM Mevastatin or knockdown of *PDE6D* or *FNTA*; $n \geq 3$ (left). A plot was derived from these data showing the loss of the BRET ratio following gene knockdowns as compared to Mevastatin treatment set to 100 % (right). Knockdown validation in (E).
(**D**) H-RasG12V-membrane anchorage BRET is not affected by knockdown of *PDE6D*; $n \geq 3$.
(**E**) Representative immunoblot data showing *PDE6D* knockdown efficiency in HEK293 EBNA cells; $n \geq 3$. Immunoblot data showing *FNTA* knockdown efficiency in HEK293 EBNA cells were previously reported by us [1].
(**F**) Assessment of K-RasG12V-membrane anchorage by BRET following FTI-277, GGTI-298, Mevastatin, Sildenafil, Deltaflexin3 or indicated combination treatments; $n \geq 2$.

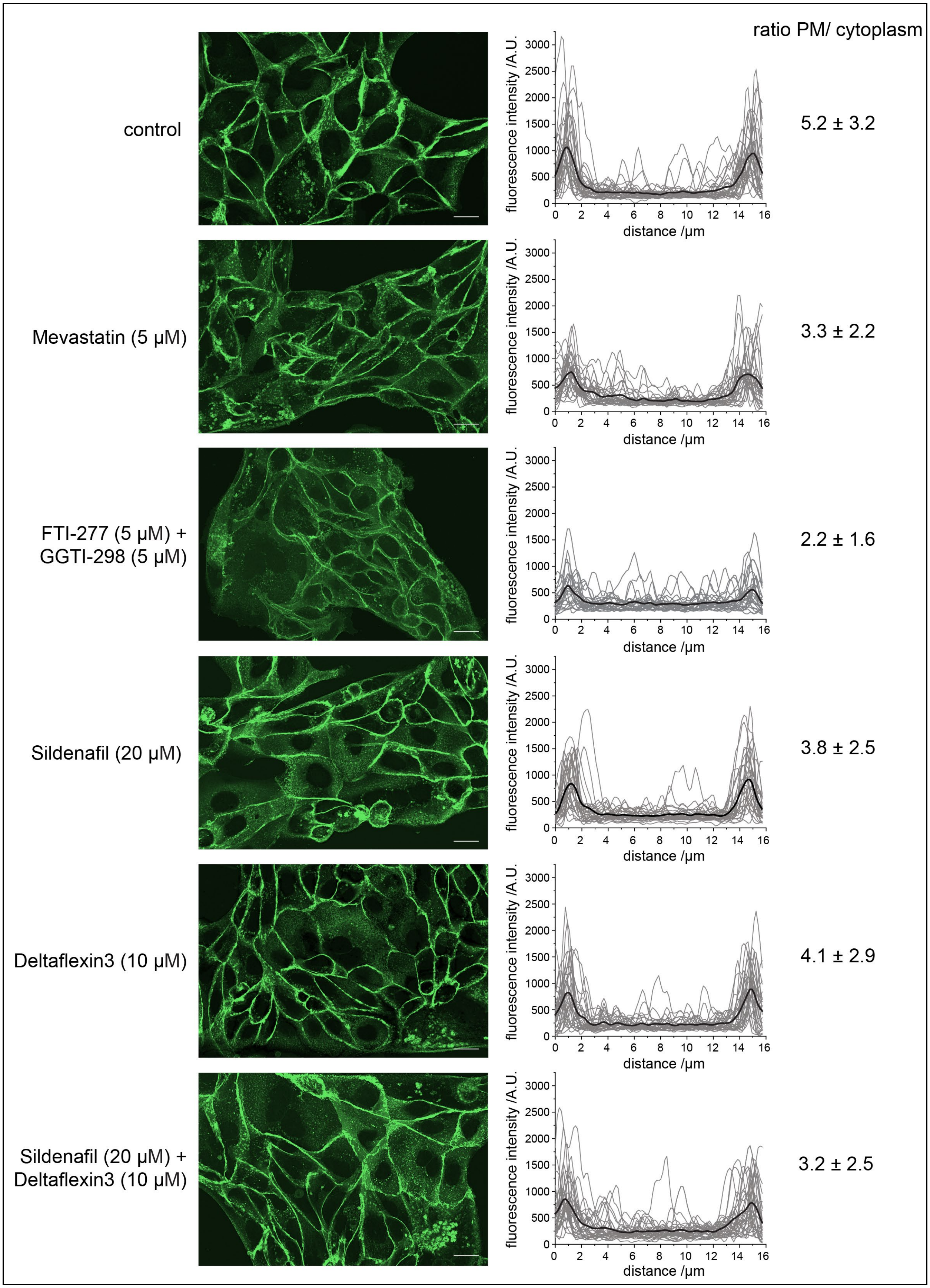
ratio PM/ cytoplasm
control
5.2 ± 3.2
Mevastatin (5 µM)
3.3 ± 2.2
FTI-277 (5 µM) +
GGTI-298 (5 µM)
2.2 ± 1.6
Sildenafil (20 µM)
3.8 ± 2.5
Deltaflexin3 (10 µM)
4.1 ± 2.9
Sildenafil (20 µM) +
Deltaflexin3 (10 µM)
3.2 ± 2.5
fluorescence intensity /A.U.
distance /µm

Figure S2. Confocal microscopy-based analysis of K-RasG12V plasma membrane localization after treatment, supplementing main Figure 2.

Left side, confocal micrographs of live MDCK cells stably expressing EGFP-K-RasG12V after a 4 h treatment with the control DMSO 0.1 %, or indicated concentrations of Mevastatin, FTI-277 and GGTI-298, Sildenafil, Deltaflexin3 or a combination of Deltaflexin3 and Sildenafil. Data are representative of three independent experiments. Image acquisition was performed using an Andor benchtop confocal microscope (Oxford Instruments, Belfast, UK) with a × 40 objective and image deconvolution applied. Image analysis was done using ImageJ software (National Institute of Health, Bethesda, MD, USA). Scale bars, 20 μm. **Right side**, intensity profile plots and quantification of the EGFP-K-RasG12V signal. Individual cell traces in gray, the mean is plotted in black ($n \geq 30$ cells per condition; plot drawn with the OriginPro software; A.U., arbitrary units). The plasma membrane (PM)/ cytoplasm ratio was determined as the ratio of the mean of the 2 peak values corresponding to plasma membrane localization and the mean of the 3 lowest dispersed plateau values corresponding to cytoplasmic localization. A decrease of this ratio corresponds to a loss EGFP-K-RasG12V plasma membrane localization. Note that the high errors associated with this analysis and the high noise in the source data (gray traces) would make it difficult to draw definite conclusions based on these data alone.

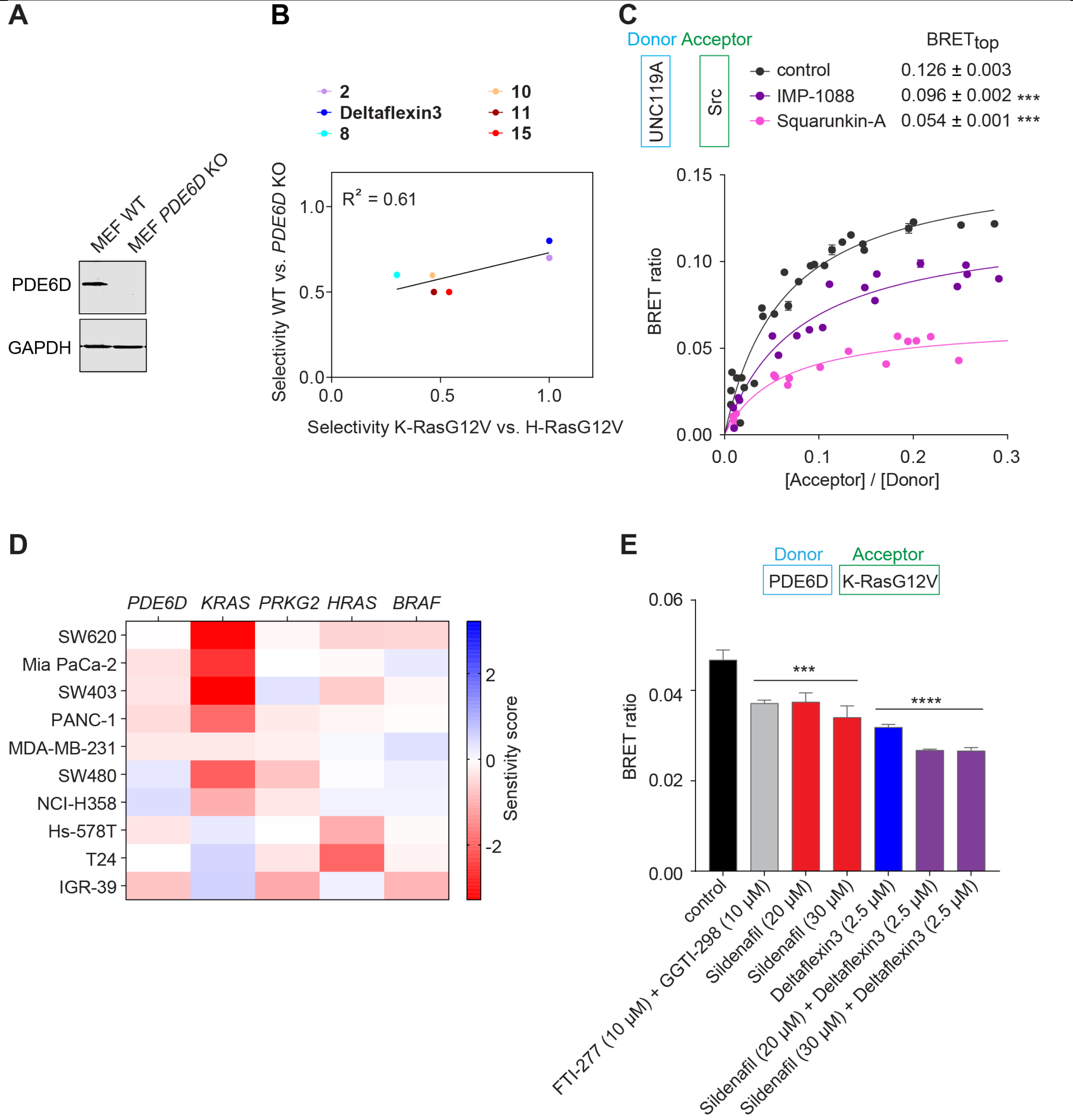

Figure S3. Data supplementing main Figures 2 - 5.

(**A**) Representative immunoblot data verifying absence of *PDE6D* in *PDE6D*-KO MEF cells.

(**B**) Correlation plot of K-RasG12V vs. H-RasG12V selectivity obtained by BRET (Figure 2B) and MEF WT vs. MEF *PDE6D* KO-viability derived PDE6D-selectivity (Figure 3A).

(**C**) BRET-titration curves of the UNC119A/ Src complex after treatment with 5 µM of the N-myristoyl-transferase-inhibitor IMP-1088 or 5 µM of the UNC119-inhibitor Squarunkin A; n ≥ 3. Statistical comparisons of BRETtop values to control were done using two-tailed Student's t-test.

(**D**) Heatmap of the ATARiS-sensitivity scores of selected genes for all cancer cell lines used in this study. Negative values indicate a decrease of proliferation upon gene knockdown and therefore a higher dependency of the cell line on that gene.
(**E**) PDE6D/ K-RasG12V BRET reduction depends on whether PDE6D or K-RasG12V is affected by indicated inhibitors. FTI-277 + GGTI-298 and Sildenafil impact on K-RasG12V, which is present in excess, due to the biosensor transfection ratio, leading to a lesser response than Deltaflexin3 blocking PDE6D. This effectively corresponds to two dynamic ranges for the two targets mapped onto the absolute BRET ratio scale of the biosensor. Thus, the potentiating effect of the combination treatments as compared to Deltaflexin3 alone can be recognized; $n \geq 2$.

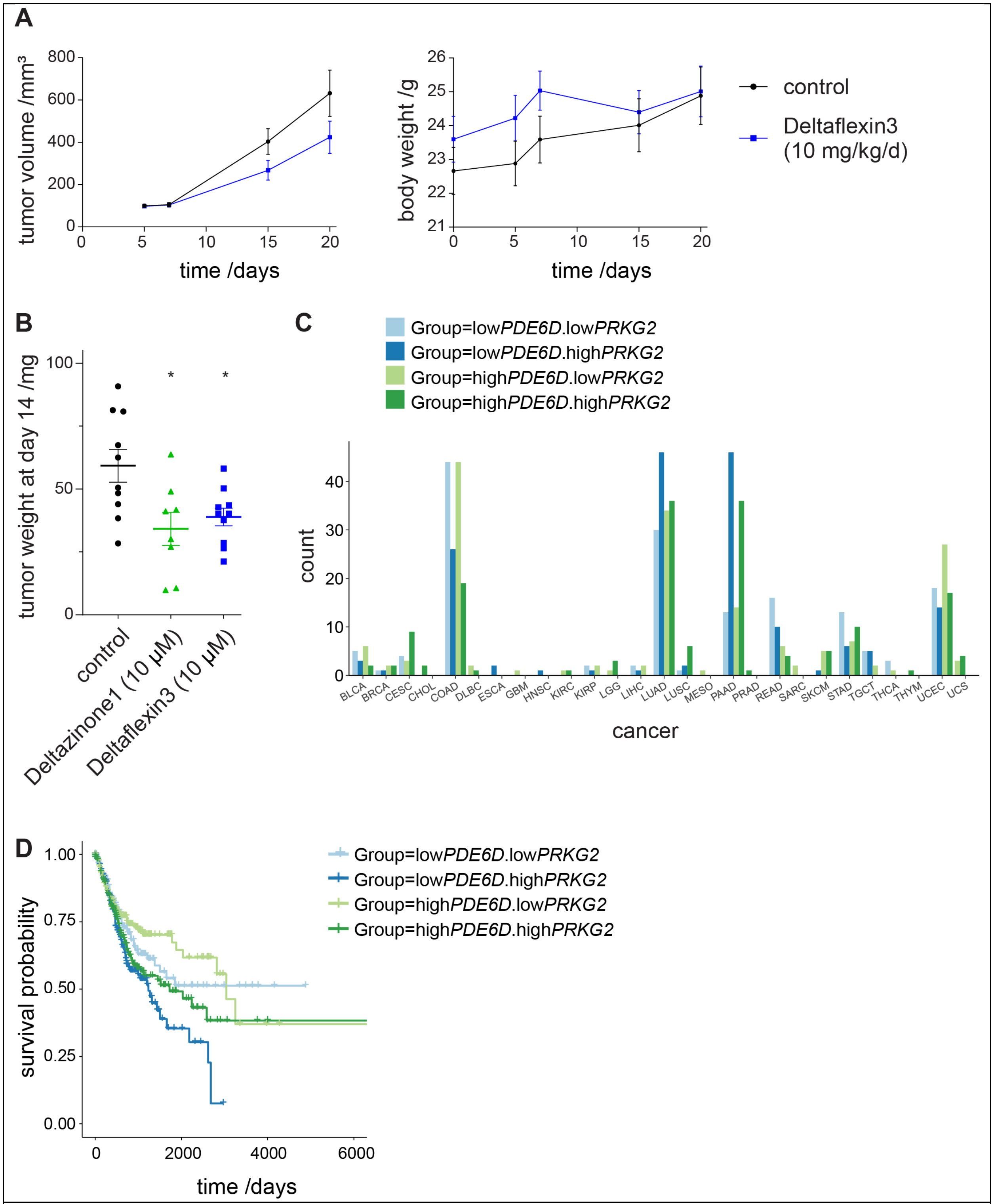

Figure S4. Data supplementing main Figure 6.

(**A**) Results of xenograft experiments of MDA-MB-231 in mice. Tumor volume (left) and body weight (right) development over time during daily treatment with vehicle control or Deltaflexin3. Per treatment group, n = 10 animals were analyzed.

(**B**) Microtumor growth CAM-assay with MDA-MB-231 cell grafts (≥ 8 per condition from n = 3). Weights of microtumors after 6 d of growth on CAM with 5 d of treatment with indicated compounds.
(**C**) Number of *KRAS* mutant patient tumor samples with indicated high or low gene expression level combinations of *PDE6D* and *PRKG2* (gene for PKG2) by cancer type (TCGA study abbreviations).
(**D**) Overall survival of *KRAS* mutant patient tumor samples from TCGA with indicated high or low gene expression level combinations of *PDE6D* and *PRKG2* (gene for PKG2). Number of patients per group: 157 (low*PDE6D*.low*PRKG2*), 165 (low*PDE6D*.high*PRKG2*), 166 (high*PDE6D*.low*PRKG2*), 159 (high*PDE6D*.high*PRKG2*). Kaplan-Meyer test for the difference between low*PDE6D*.high*PRKG2* and high*PDE6D*.low*PRKG2* groups: p =0.0006.

Table S1: Materials and Equipment used in the study

REAGENT or RESOURCE	SOURCE	IDENTIFIER
Antibodies		
Phospho-p44/42 MAPK (Erk1/2) (Thr202/Tyr204) (E10) Mouse mAb	Cell Signaling Technology	Cat#9106 RRID:AB_331768
p44/42 MAPK (Erk1/2) Rabbit pAb	Cell Signaling Technology	Cat#9102 RRID:AB_330744)
Phospho-S6 Ribosomal Protein (Ser235/236) (D57.2.2E) XP Rabbit mAb	Cell Signaling Technology	Cat#4858 RRID:AB_916156
S6 Ribosomal Protein (54D2) Mouse mAb	Cell Signaling Technology	Cat#2317 RRID:AB_2238583
Phospho-MEK1/2 (Ser217/221) (41G9) Rabbit mAb	Cell Signaling Technology	Cat#9154 RRID:AB_2138017
MEK1/2 (L38C12) Mouse mAb	Cell Signaling Technology	Cat#4694 RRID:AB_10695868
Phospho-Akt (Ser473) (D9E) XP® Rabbit mAb	Cell Signaling Technology	Cat#4060 RRID:AB_2315049
Akt (pan) (40D4) Mouse mAb	Cell Signaling Technology	Cat#2920 RRID:AB_1147620
Mouse monoclonal anti-β-actin clone AC-15	Sigma-Aldrich	Cat#A5441 RRID:AB_476744
Mouse monoclonal PDE6D (E-7)	Santa Cruz Biotechnology	Cat#sc-166854 RRID:AB_2161460
Rabbit polyclonal anti-GAPDH	Sigma-Aldrich	Cat#G9545, RRID:AB_796208
IRDye 680RD goat anti-rabbit IgG	LI-COR Biosciences	Cat#926-68071, RRID:AB_10956166
IRDye 800CW donkey anti-mouse IgG	LI-COR Biosciences	Cat#926-32212, RRID:AB_621847
Bacterial and virus strains		
E. coli DH10B	New England Biolabs	Cat#C3019I
E. coli BL21 Star (DE3)pLysS	Thermo Fisher Scientific	Cat#C602003
Biological samples		
N/A	N/A	N/A
Chemicals, peptides, and recombinant proteins		

Fluorescein-labelled Atorvastatin (F-Ator)	Piramal Pharma Solutions custom synthesis as in [2]	N/A
Fluorescein-labelled Rheb (F-Rheb)	Described in [3]	N/A
Benzethonium chloride	Sigma-Aldrich	Cat#53751-50G; CAS121-54-0
AMG 510	MedChem Express	Cat#HY-114277; CAS2296729-00-3
ARS-1620	MedChem Express	Cat#HY-U00418; CAS1698055-85-4
FTI-277 hydrochloride	VWR chemicals	Cat#BIOV2874-5; CAS180977-34-8
GGTI-298	Selleck Chemicals	Cat#S7466; CAS1217457-86-7
Deltazinone1	Piramal Pharma Solutions custom synthesis as in [4]	N/A
Deltarasin	Selleck Chemicals	Cat#S7224; CAS1440898-61-2
Deltasonamide1	Piramal Pharma Solutions custom synthesis as in [5]	N/A
Mevastatin	Alfa Aesar by Thermo Fisher Scientific	Cat#J61357.MB; CAS73573-88-3
Trametinib	MedChem Express	Cat#SC-364639; CAS871700-17-3
Vemurafenib (PLX4032, RG7204)	Selleck Chemicals	Cat#S1267; CAS918504-65-1
Squarunkin A	Axon Medchem	Cat#2778; CAS2101958-02-3
IMP-1088	Cayman Chemicals	Cat#25366-1; CAS2059148-82-0
Atorvastatin (calcium salt hydrate)	Cayman Chemicals	Cat#10493; CAS357164-38-6
Sildenafil	MedChem Express	Cat#38756; CAS139755-83-2
Tadalafil	MedChem Express	Cat#HY-90009A; CAS171596-29-5
Deltaflexin-2	[6]	N/A
1	This paper	N/A
2	This paper	N/A
3	This paper	N/A
4 (Deltaflexin3)	This paper	N/A
5	This paper	N/A

6	This paper	N/A
7	This paper	N/A
8	This paper	N/A
9	This paper	N/A
10	This paper	N/A
11	This paper	N/A
12	This paper	N/A
13	This paper	N/A
14	This paper	N/A
15	This paper	N/A
16	This paper	N/A
Critical commercial assays		
Gateway LR Clonase II enzyme mix	Thermo Fisher Scientific	Cat#11791020
jetPRIME transfection reagent	Polyplus	Cat#101000046
Lipofectamine 2000 transfection reagent	Thermo Fisher Scientific	Cat#11668019
Coelenterazine 400a; 2,8-Dibenzyl-6-phenyl-imidazo[1,2a]pyrazin-3-(7H)-one; DeepBlueC	Gold Biotechnology	Cat#C-320-1
alamarBlue cell viability reagent	Thermo Fisher Scientific	Cat#DAL1100
Experimental models: Cell lines		
Human cell line, HEK293-EBNA (HEK)	Prof. Florian M. Wurm, EPFL	RRID:CVCL_6974
Human cell line, SW620	ATCC	CCL-227, RRID:CVCL_0547
Human cell line, MIA PaCa-2	ATCC	CRM-CRL-1420, RRID:CVCL_0428
Human cell line, cell line, PANC-1	ATCC	CRL-1469, RRID:CVCL_0480
Human cell line, MDA-MB-231	ATCC	HTB-26, RRID:CVCL_0062
Human cell line, NCI-H358	ATCC	CRL-5807, RRID:CVCL_1559
Human cell line, SW480	DSMZ	ACC-313, RRID:CVCL_0546
Human cell line, Hs 578T	DSMZ	ACC 781, RRID:CVCL_0332
Human cell line, T24	DSMZ	ACC 376, RRID:CVCL_0554
Human cell line, IGR-39	DSMZ	ACC 239, RRID:CVCL_2076
Human cell line, SW403	ATCC	CCL-230 RRID:CVCL_0545

Mouse cell line, WT MEF cells	ATCC	CRL-2991, RRID:CVCL_L690
Mouse cell line, *PDE6D* KO MEF cells	Prof. Richard A. Kahn, Emory University School of Medicine	N/A
Experimental models: Organisms/strains		
SPF eggs	VALO BioMedia GmbH	N/A
Female athymic nude mice (Hsd:Athymic Nude-Foxn1)	ENVIGO RMS SARL	N/A
Oligonucleotides		
ON-TARGETplus SMARTpool siRNA Human PDE6D 5147 4 targets	DHARMACON	Cat#L-004310-00-0005
ON-TARGETplus SMARTpool siRNA mouse PDE6d 4 targets	DHARMACON	Cat#L-062279-01-0005
Hs_FNTA_6 CCGGGATGCTATTGAGTTAAA	QIAGEN	Cat#SI02661995
Negative Control siRNA AATTCTCCGAACGTGTCACGT	QIAGEN	Cat#1027310
Recombinant DNA		
C413-E36_CMV promoter	[7]	Addgene, #162927
C453-E04_CMV promoter	[7]	Addgene, #162973
pDest-305	[7]	Addgene, #161895
pDest-312	[7]	Addgene, #161897
C231-E13_Rluc8-stop	[7]	Addgene, FNL Combinatorial Cloning Platform, kit #1000000211
C511-E03_Rluc8-no stop	[7]	Addgene, FNL Combinatorial Cloning Platform, kit #1000000211
pDONR235-GFP2_stop	[8]	N/A
pDONR257-GFP2_no stop	[8]	N/A
Hs. KRas4B G12V	RAS mutant collection V2.0, Ras-Initiative	Addgene, #83132
Hs. HRas G12V	RAS mutant collection V2.0, Ras-Initiative	Addgene, #83184
Hs. PDE6D	R3 RAS Pathway Clone Collection #1, Ras-Initiative	#R702-E30

Hs. Kras4B C185S	RAS mutant collection V2.0, Ras-Initiative	#83139
Hs. UNC119A (NM_005417.4, without stop codon)	Genecust	N/A
Hs. Src (NM_005148.4, without stop codon)	Genecust	N/A
pDest305-CMV-GFP2- K-Ras4BG12V	[8]	N/A
pDest305-CMV-Rluc8- K-Ras4BG12V	[8]	N/A
pDest305-CMV-GFP2- H-RasG12V	[8]	N/A
pDest305-CMV-Rluc8- H-RasG12V	[8]	N/A
pDest312-CMV-Rluc8- PDE6D	This paper	N/A
pDest312-CMV-UNC119A-Rluc8	This paper	N/A
pDest312-CMV-SRC-GFP2	This paper	N/A
pDest305-CMV-GFP2- K-Ras4BG12V-C185S	This paper	N/A
pcDNA3.1(+)	Invitrogen	#V79020
pDest-His6-MBP-PDE6D	Ras-Initiative	#R702-X31-566
Software and algorithms		
Maestro	Schrödinger Release 2019-2; Maestro, Schrödinger, LLC: New York, NY, USA, 2019.	https://www.schrodinger.com/products/maestro
Glide	[9]	https://www.schrodinger.com/products/glide
OPLS3	[10]	https://www.schrodinger.com/products/opls4
VSGB 2.0 solvation model	[11]	https://doi.org/10.1002/prot.23106
SeeSAR v10.3	BioSolveIT GmbH	https://www.biosolveit.de/SeeSAR
OpenBabel v2.3.2	[12]	http://openbabel.org/
BREEZE pipeline	[13]	https://breeze.fimm.fi/
SynergyFinder v3.0	[14]	https://synergyfinder.fimm.fi/
Project DRIVE	[15]	https://oncologynibr.shinyapps.io/drive/
MARS Data Analysis Software	BMG LABTECH	https://www.bmglabtech.com/en/microplate-reader-software/
R v4.2.1	[16]	https://www.r-project.org/

GraphPad Prism v9.5.1	GraphPad by Dotmatics,	https://www.graphpad.com/
ImageJ	[17]	https://imagej.net/
OriginPro	OriginLab Corporation	https://www.originlab.com/
Other		
CLARIOstar Plus Microplate Reader	BMG LABTECH	https://www.bmglabtech.com/en/clariostar-plus/
Odyssey CLx Infrared Imaging System	LI-COR Biosciences	https://www.licor.com/bio/odyssey-clx/
ÄKTA pure chromatography system	Cytiva	https://www.cytivalifesciences.com/en/us/shop/chromatography/chromatography-systems/akta-pure-p-05844
Elmasonic S 40 H	Elma	https://www.elma-ultrasonic.com/

Supporting Information References

(1) Manoharan, G. B.; Laurini, C.; Bottone, S.; Ben Fredj, N.; Abankwa, D. K. K-Ras Binds Calmodulin-Related Centrin1 with Potential Implications for K-Ras Driven Cancer Cell Stemness. *Cancers (Basel)* **2023**, *15* (12). DOI: 10.3390/cancers15123087.

(2) Zimmermann, G.; Papke, B.; Ismail, S.; Vartak, N.; Chandra, A.; Hoffmann, M.; Hahn, S. A.; Triola, G.; Wittinghofer, A.; Bastiaens, P. I.; Waldmann, H. Small molecule inhibition of the KRAS-PDEdelta interaction impairs oncogenic KRAS signalling. *Nature* **2013**, *497* (7451), 638-642, 10.1038/nature12205. DOI: 10.1038/nature12205.

(3) Ismail, S. A.; Chen, Y. X.; Rusinova, A.; Chandra, A.; Bierbaum, M.; Gremer, L.; Triola, G.; Waldmann, H.; Bastiaens, P. I.; Wittinghofer, A. Arl2-GTP and Arl3-GTP regulate a GDI-like transport system for farnesylated cargo. *Nat Chem Biol* **2011**, *7* (12), 942-949. DOI: 10.1038/nchembio.686.

(4) Papke, B.; Murarka, S.; Vogel, H. A.; Martin-Gago, P.; Kovacevic, M.; Truxius, D. C.; Fansa, E. K.; Ismail, S.; Zimmermann, G.; Heinelt, K.; Schultz-Fademrecht, C.; Al Saabi, A.; Baumann, M.; Nussbaumer, P.; Wittinghofer, A.; Waldmann, H.; Bastiaens, P. I. Identification of pyrazolopyridazinones as PDEdelta inhibitors. *Nat Commun* **2016**, *7*, 11360, 10.1038/ncomms11360. DOI: 10.1038/ncomms11360.

(5) Martin-Gago, P.; Fansa, E. K.; Klein, C. H.; Murarka, S.; Janning, P.; Schurmann, M.; Metz, M.; Ismail, S.; Schultz-Fademrecht, C.; Baumann, M.; Bastiaens, P. I.; Wittinghofer, A.; Waldmann, H. A PDE6delta-KRas Inhibitor Chemotype with up to Seven H-Bonds and Picomolar Affinity that Prevents Efficient Inhibitor Release by Arl2. *Angew Chem Int Ed Engl* **2017**, *56* (9), 2423-2428. DOI: 10.1002/anie.201610957.

(6) Siddiqui, F. A.; Alam, C.; Rosenqvist, P.; Ora, M.; Sabt, A.; Manoharan, G. B.; Bindu, L.; Okutachi, S.; Catillon, M.; Taylor, T.; Abdelhafez, O. M.; Lonnberg, H.; Stephen, A. G.; Papageorgiou, A. C.; Virta, P.; Abankwa, D. PDE6D Inhibitors with a New Design Principle Selectively Block K-Ras Activity. *ACS Omega* **2020**, *5* (1), 832-842, 10.1021/acsomega.9b03639. DOI: 10.1021/acsomega.9b03639.

(7) Wall, V. E.; Garvey, L. A.; Mehalko, J. L.; Procter, L. V.; Esposito, D. Combinatorial assembly of clone libraries using site-specific recombination. *Methods Mol Biol* **2014**, *1116*, 193-208. DOI: 10.1007/978-1-62703-764-8_14 From NLM Medline.

(8) Okutachi, S.; Manoharan, G. B.; Kiriazis, A.; Laurini, C.; Catillon, M.; McCormick, F.; Yli-Kauhaluoma, J.; Abankwa, D. A Covalent Calmodulin Inhibitor as a Tool to Study Cellular Mechanisms of K-Ras-Driven Stemness. *Front Cell Dev Biol* **2021**, *9*, 665673. DOI: 10.3389/fcell.2021.665673 From NLM PubMed-not-MEDLINE.

(9) Friesner, R. A.; Murphy, R. B.; Repasky, M. P.; Frye, L. L.; Greenwood, J. R.; Halgren, T. A.; Sanschagrin, P. C.; Mainz, D. T. Extra precision glide: docking and scoring incorporating a model of hydrophobic enclosure for protein-ligand complexes. *J Med Chem* **2006**, *49* (21), 6177-6196, 10.1021/jm051256o. DOI: 10.1021/jm051256o.

(10) Harder, E.; Damm, W.; Maple, J.; Wu, C.; Reboul, M.; Xiang, J. Y.; Wang, L.; Lupyan, D.; Dahlgren, M. K.; Knight, J. L.; Kaus, J. W.; Cerutti, D. S.; Krilov, G.; Jorgensen, W. L.; Abel, R.; Friesner, R. A. OPLS3: A Force Field Providing Broad Coverage of Drug-like Small Molecules and Proteins. *J Chem Theory Comput* **2016**, *12* (1), 281-296, 10.1021/acs.jctc.5b00864. DOI: 10.1021/acs.jctc.5b00864.

(11) Li, J.; Abel, R.; Zhu, K.; Cao, Y.; Zhao, S.; Friesner, R. A. The VSGB 2.0 model: a next generation energy model for high resolution protein structure modeling. *Proteins* **2011**, *79* (10), 2794-2812. DOI: 10.1002/prot.23106 From NLM Medline.

(12) O'Boyle, N. M.; Banck, M.; James, C. A.; Morley, C.; Vandermeersch, T.; Hutchison, G. R. Open Babel: An open chemical toolbox. *J Cheminform* **2011**, *3*, 33. DOI: 10.1186/1758-2946-3-33.

(13) Potdar, S.; Ianevski, A.; Mpindi, J. P.; Bychkov, D.; Fiere, C.; Ianevski, P.; Yadav, B.; Wennerberg, K.; Aittokallio, T.; Kallioniemi, O.; Saarela, J.; Ostling, P. Breeze: an integrated quality control and data analysis application for high-throughput drug screening. *Bioinformatics* **2020**, *36* (11), 3602-3604. DOI: 10.1093/bioinformatics/btaa138.

(14) Ianevski, A.; Giri, A. K.; Aittokallio, T. SynergyFinder 3.0: an interactive analysis and consensus interpretation of multi-drug synergies across multiple samples. *Nucleic Acids Res* **2022**, *50* (W1), W739-W743. DOI: 10.1093/nar/gkac382 From NLM Medline.

(15) McDonald Iii, E. R.; de Weck, A.; Schlabach, M. R.; Billy, E.; Mavrakis, K. J.; Hoffman, G. R.; Belur, D.; Castelletti, D.; Frias, E.; Gampa, K.; Golji, J.; Kao, I.; Li, L.; Megel, P.; Perkins, T. A.; Ramadan, N.; Ruddy, D. A.; Silver, S. J.; Sovath, S.; Stump, M.; Weber, O.; Widmer, R.; Yu, J.; Yu, K.; Yue, Y.; Abramowski, D.; Ackley, E.; Barrett, R.; Berger, J.; Bernard, J. L.; Billig, R.; Brachmann, S. M.; Buxton, F.; Caothien, R.; Caushi, J. X.; Chung, F. S.; Cortés-Cros, M.; deBeaumont, R. S.; Delaunay, C.; Desplat, A.; Duong, W.; Dwoske, D. A.; Eldridge, R. S.; Farsidjani, A.; Feng, F.; Feng, J.; Flemming, D.; Forrester, W.; Galli, G. G.; Gao, Z.; Gauter, F.; Gibaja, V.; Haas, K.; Hattenberger, M.; Hood, T.; Hurov, K. E.; Jagani, Z.; Jenal, M.; Johnson, J. A.; Jones, M. D.; Kapoor, A.; Korn, J.; Liu, J.; Liu, Q.; Liu, S.; Liu, Y.; Loo, A. T.; Macchi, K. J.; Martin, T.; McAllister, G.; Meyer, A.; Mollé, S.; Pagliarini, R. A.; Phadke, T.; Repko, B.; Schouwey, T.; Shanahan, F.; Shen, Q.; Stamm, C.; Stephan, C.; Stucke, V. M.; Tiedt, R.; Varadarajan, M.; Venkatesan, K.; Vitari, A. C.; Wallroth, M.; Weiler, J.; Zhang, J.; Mickanin, C.; Myer, V. E.; Porter, J. A.; Lai, A.; Bitter, H.; Lees, E.; Keen, N.; Kauffmann, A.; Stegmeier, F.; Hofmann, F.; Schmelzle, T.; Sellers, W. R. Project DRIVE: A Compendium of Cancer Dependencies and Synthetic Lethal Relationships Uncovered by Large-Scale, Deep RNAi Screening. *Cell* **2017**, *170* (3), 577-586.e510, 10.1016/j.cell.2017.07.005. DOI: papers3://publication/doi/10.1016/j.cell.2017.07.005.

(16) R, C. T. R: A Language and Environment for Statistical Computing. **2022**.

(17) Schindelin, J.; Arganda-Carreras, I.; Frise, E.; Kaynig, V.; Longair, M.; Pietzsch, T.; Preibisch, S.; Rueden, C.; Saalfeld, S.; Schmid, B.; Tinevez, J. Y.; White, D. J.; Hartenstein, V.; Eliceiri, K.; Tomancak, P.; Cardona, A. Fiji: an open-source platform for biological-image analysis. *Nat Methods* **2012**, *9* (7), 676-682. DOI: 10.1038/nmeth.2019 From NLM Medline.
